# Inhibition of HSP90β Improves Lipid Disorders by Promoting Mature SREBPs Degradation via the Ubiquitin-proteasome System

**DOI:** 10.7150/thno.36505

**Published:** 2019-08-12

**Authors:** Zu-Guo Zheng, Xin Zhang, Xiao-Xiao Liu, Xiu-Xiu Jin, Lunzhi Dai, Hui-Min Cheng, Dan Jing, Pyone Myat Thu, Mu Zhang, Hongyang Li, Jing Zhu, Chang Liu, Bin Xue, Yu Li, Ligong Chen, Cheng Peng, Weiliang Zhu, Lin Wang, Junli Liu, Hui-Jun Li, Ping Li, Xiaojun Xu

**Affiliations:** 1 State Key Laboratory of Natural Medicines, China Pharmaceutical University, 210009, Nanjing, Jiangsu, China.; 2 Jiangsu Key Laboratory of Drug Discovery for Metabolic Diseases, China Pharmaceutical University, 210009, Nanjing, Jiangsu, China.; 3 Shanghai Diabetes Institute, Department of Endocrinology and Metabolism, Shanghai Jiao Tong University Affiliated Sixth People's Hospital, 200233, Shanghai, China.; 4 Department of General Practice and Lab of PTM, State Key Laboratory of Biotherapy, West China Hospital, Sichuan University, and Collaborative Innovation Center of Biotherapy, Chengdu 610041, China.; 5 Institute of Dermatology, Chinese Academy of Medical Sciences, Peking Union Medical College. 210009, Nanjing, Jiangsu, China.; 6 Department of Infection Diseases, Hangzhou First People's Hospital, 310006, Zhejiang, China.; 7 State Key Laboratory of Pharmaceutical Biotechnology and Jiangsu Key Laboratory of Molecular Medicine and School of Medicine, Nanjing University, 210093, Nanjing, Jiangsu, China.; 8 Key Laboratory of Nutrition and Metabolism, Institute for Nutritional Sciences, Shanghai Institutes for Biological Sciences, Chinese Academy of Sciences, University of Chinese Academy of Sciences, 200031, Shanghai, China.; 9 School of Pharmaceutical Sciences, Tsinghua University, Beijing, 100084.; 10 Drug Discovery and Design Center, CAS Key Laboratory of Receptor Research, Shanghai Institute of Materia Medica, Chinese Academy of Sciences, 555 Zuchongzhi Road, Shanghai, 201203, China.; 11 Department of Hepatobiliary Surgery, Xi-Jing Hospital, Fourth Military Medical University, Xi'an, 710032, China.

**Keywords:** HSP90β, lipid metabolism, corylin, SREBPs, proteasomal degradation

## Abstract

**Rationale:** Heat shock protein 9 (HSP90) are a family of the most highly expressed cellular proteins and attractive drug targets against cancer, neurodegeneration diseases, etc. HSP90 proteins have also been suggested to be linked to lipid metabolism. However, the specific function of HSP90 paralogs, as well as the underlying molecular cascades remains largely unknown. This study aims to unravel the paralog-specific role of HSP90 in lipid metabolism and try to discover paralog-specific HSP90 inhibitors.

**Methods:** In non-alcohol fatty liver disease (NAFLD) patients, as well as in diet induced obese (DIO) mice, expression of HSP90 paralogs were analyzed by immunohistochemistry and western blot. In hepatocytes and in DIO mice, HSP90 proteins were knockdown by siRNAs/shRNAs, metabolic parameters, as well as downstream signaling were then investigated. By virtue screening, corylin was found to bind specifically to HSP90β. Using photo-affinity labeling and mass spectrum, corylin binding proteins were identified. After oral administration of corylin, its lipid lowering effects in different metabolic disease mice models were evaluated.

**Results:** We showed that hepatic HSP90β, rather than HSP90α, was overexpressed in NAFLD patients and obese mice. Hepatic HSP90β was also clinical relevant to serum lipid level. Depletion of HSP90β promoted mature sterol regulatory element-binding proteins (mSREBPs) degradation through Akt-GSK3β-FBW7 pathway, thereby dramatically decreased the content of neutral lipids and cholesterol. We discovered an HSP90β-selective inhibitor (corylin) that only bound to its middle domain. We found that corylin treatment partially suppressed Akt activity only at Thr308 site and specifically promoted mSREBPs ubiquitination and proteasomal degradation. Corylin treatment significantly reduced lipid content in both liver cell lines and human primary hepatocytes. In animal studies, we showed that corylin ameliorated obesity-induced fatty liver disease, type 2 diabetes and atherosclerosis.

**Principle conclusions:** HSP90β plays a parolog-specific role in regulating lipid homeostasis. Compound that selectively inhibits HSP90β could be useful in the clinic for the treatment for metabolic diseases.

## Introduction

Heat shock protein 90 (HSP90) is a molecular chaperone family member that is crucial for the correct folding of many newly synthesized peptides and rematuration of denatured/misfolded client proteins. The client proteins of HSP90 are intensively involved in physiological, pathological and evolutionary processes 1. Inhibition of HSP90 activity by small-molecule inhibitors has been demonstrated to cause potent reversal of diseases in the model of cancer, neurodegenerative disorders, epilepsy, infections and inflammatory diseases 2. Although the role of HSP90 in metabolic diseases has gradually aroused people's concern 3-5, the underlying detailed mechanism, as well as the paralog specific roles remain elusive. The HSP90 family consists of four paralogs: *HSP90AA1, HSP90AB1, HSP90B1* (encoding glucose-regulated protein 94), and *TRAP1* (encoding TNF receptor-associated protein-1) 6. Genetic evidences already suggest functional difference between HSP90α and HSP90β 7. However, due to common location 8 and the high sequence identity between these two paralogs (85.8% identity and 93.4% similarity of amino acid sequence), it is rather difficult to distinguish their functional specialty through biological separation. Most of the pan-HSP90 inhibitors bind to the highly conserved N-terminal ATP binding domain (NTD). As a consequence, these pan-HSP90 inhibitors bind both HSP90α and HSP90β 9. To interrogate the different functions of HSP90α and HSP90β, there is pressing need for the discovery of paralog-specific inhibitors.

Lipids are essential for energy homeostasis, reproductive and organ physiology and numerous aspects of cellular biology 10. Dysregulation of lipid homeostasis leads to metabolic diseases, including obesity, type 2 diabetes, non-alcoholic fatty liver disease (NAFLD) and cardiovascular diseases 11, 12. Elevated peripheral non-esterified fatty acid (NEFA) pool and hepatic *de novo* lipogenesis (DNL) are key factors that contribute the lipid accumulation in the liver. Notably, in patients with non-alcoholic fatty liver disease (NAFLD), triglyceride turnover rates were not increased 13, while DNL accounts more than 20% of liver TG, 3-5 times higher than healthy populations 14, 15. Thus, targeting DNL is a potent strategy to develop medications treating metabolic diseases 16, 17. Sterol regulatory element-binding proteins (SREBPs) are major regulators of DNL that control the expression of genes involved in the biosynthesis and uptake of fatty acids, triglycerides, cholesterol and phospholipids 18, 19. Even though a previous report has already shown that inhibition of HSP90 by 17-AAG modulates lipid homeostasis *via* SREBP pathway 20, the clinical and mechanical relevance between HSP90 paralogs and lipid disorders remains largely elusive. Moreover, 17-AAG, as a pan-inhibitor of HSP90, has a wide range of effects on their clients and shows some serious side effects, such as primarily diarrhea, fatigue and hepatotoxicity 21. This makes 17-AAG unsuitable for long-term treatment of chronic diseases, such as metabolic diseases. Therefore, the search for HSP90 inhibitors with lower toxicity has become a new strategy for the treatment of metabolic disorders.

In this study, we found that HSP90β, rather than HSP90α, is clinical relevant to lipid dysregulation in NAFLD patients and animal models. It was demonstrated that HSP90β is vital in regulating fatty acid and cholesterol metabolism. Mechanistically, HSP90β promotes mSREBPs ubiquitination and, thus, increasing proteasomal degradation dependent on Akt-GSK3β-FBW7 pathway. Corylin, a natural product from the fruits of *Psoralea corylifolia* Linn., was identified as a novel selective HSP90β inhibitor. *In vivo*, corylin treatment reduced body weight gain and ameliorated hyperlipidemia, insulin resistance and atherosclerosis. Overall, these results suggest that inhibiting HSP90β may be a potential therapeutic treatment of lipid disorders and corylin may become a promising lead compound to treat obesity-induced fatty liver disease, type 2 diabetes and atherosclerosis.

## Materials and Methods

### Cell culture

HEK293T cell lines (293T cells) were obtained from the American Type Culture Collection. HL-7702 cells were purchased from Keygen Biotech (Nanjing, China). Both of the cell lines were grown in medium B under 37 °C, 5% CO_2_. HH (Human Hepatocytes) cells were purchased from ScienCell Research Laboratories (San Diego, CA, USA) and were cultured in 10 cm^2^ flask coated with poly-L-lysine in medium E.

### Animal experiments

The laboratory animal facility in the animal experimental center has been accredited by Association for Assessment and Accreditation of Laboratory Animal Care International. All experiments and animal care in this study were conducted in accordance with the national and international directives (the Provision and General Recommendation of Chinese Experimental Animals Administration Legislation and Guide for the Care and Use of Laboratory Animals, United States National Research Council, 2011) and approved by the Science and Technology Department of Jiangsu Province (SYXK (SU) 2016-0011). The C57BJ/6L and ApoE^-/-^ mice (SPF grade, six weeks old, 20~24 g) were purchased from Nanjing University (Nanjing, China). The animals were kept under a consistent temperature (24 °C) with 12 h light/dark cycle and fed standard food pellets with access to sterile water *ad libitum*. HFD was contained 60% fat, 20.6% carbohydrate and 19.4% protein w/w. WD for lipid disorder was contained 20% fat, 1.25% cholesterol, 0.5% cholic acid w/w. WD for atherosclerosis contained 60% fat, 20.6% carbohydrate, 1.25% cholesterol and 19.4% protein w/w.

### Human nonalcoholic fatty liver and normal healthy liver samples

Human liver samples from patients with NAFLD who underwent liver biopsy, resection. Nonalcoholic fatty liver was diagnosed by abdominal ultrasound and was verified according to liver histology. The liver samples were from treatment naïve patients following bariatric surgery to rule out the possibility that medication could complicate the experimental results. Normal human liver tissue was the non-involved surrounding tissue, obtained from NAFLD-free donors undergoing partial hepatectomy for hepatocarcinoma. The information of NAFLD-free donors was listed in Supplementary Table 1. An informed consent in writing was obtained from each patient and the study protocol was conformed to the ethical guidelines of the 1975 Declaration of Helsinki as reflected in a priori approval by the IRB.

### Statistical analysis

All data are expressed as the means standard error (SE). Statistical significance was calculated by student's *t*-test, one-way ANOVA or two-way ANOVA. When ANOVA indicated a significant difference among the groups, the statistical difference between two groups was compared using a stricter criterion for statistical significance. Differences with p <0.05 were considered to be statistically significant (p*<0.05, p**<0.01, p***<0.001, NS=Non-significant).

### Additional methods

Detailed materials and methods are available in the supplemental data.

## Results

### The expression of HSP90β, rather than HSP90α, was upregulated in the non-alcoholic fatty liver disease

**Figure 1:**
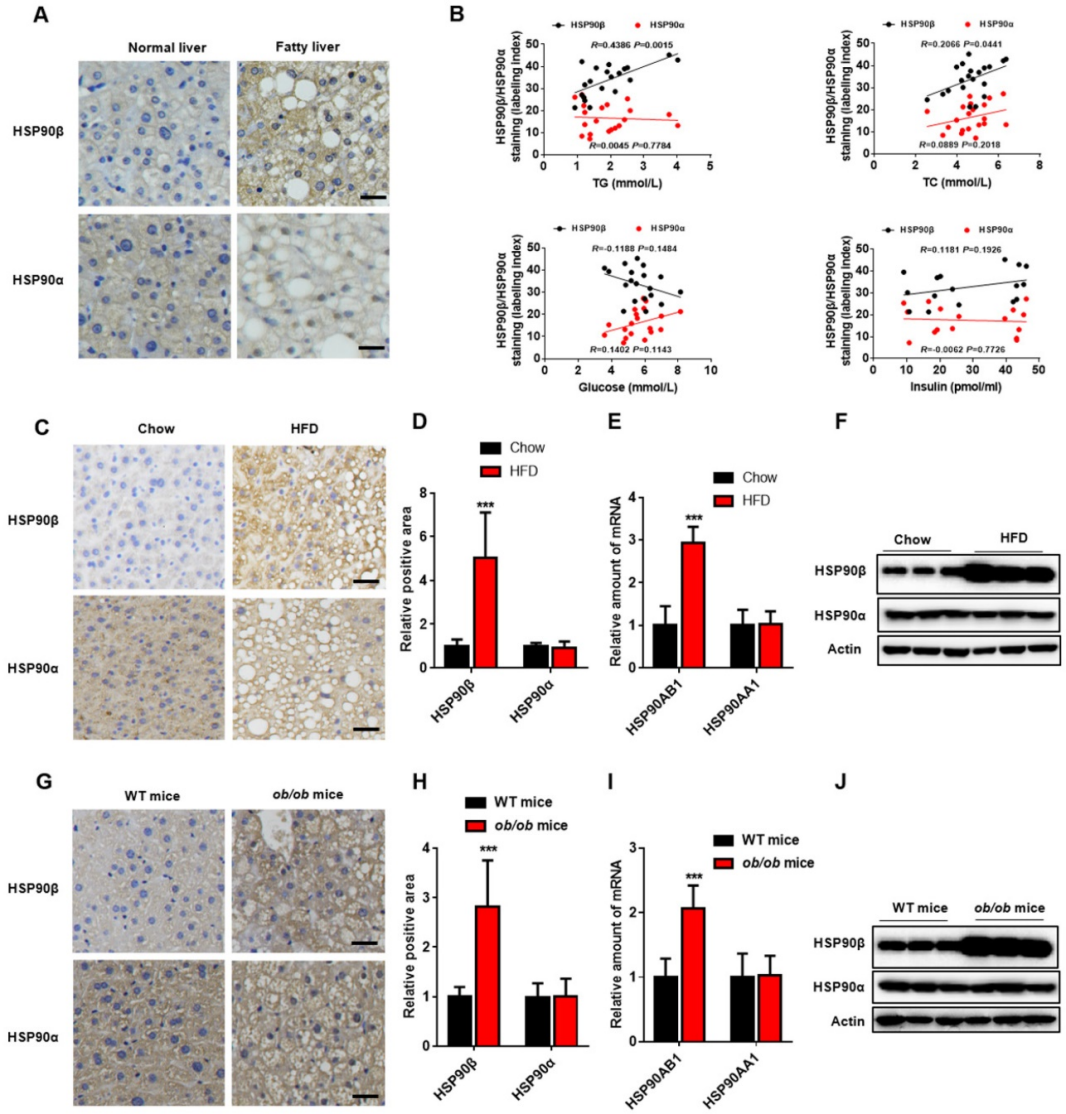
** The Level of HSP90 paralogs in the Liver of Obese Mice and NAFLD Patients. (**A) The immunohistochemistry staining of HSP90β or HSP90α in the liver of healthy donors (n=5) and NAFLD patients (n=20). (B) The correlation between immunostaining signal of HSP90s and the clinical parameters including serum level of glucose, insulin, TG and TC (n=20). (C, D, G, H) Liver sections of HFD-induced obese mice (C, D) or *ob/ob* mice (G, H) were stained with HSP90 paralog specific antibodies. (D and H) The mRNA level of *HSP90AB1* or *HSP90AA1* in the liver was analyzed in HFD- induced obese mice (E) and *ob/ob* mice (I), respectively. n = 9 for chow group or HFD group; n = 14 for WT mice group, n = 8 for *ob/ob* mice group. (F and J) The protein level of HSP90α or HSP90β in the liver was analyzed by western blot in HFD- induced obese mice (E) and *ob/ob* mice (I), respectively. Error bars are represented as mean ± SEM. The Pearson correlation coefficient is used to test the correlation between HSP90 paralogs expression and clinical parameters (B). Student *t*-test was used to analyze significance between two sets of data (D and F). ***p < 0.001 *vs* chow diet mice or WT mice.

To evaluate potential role of HSP90 in the pathogenesis of lipid metabolic disorders, we measured the hepatic HSP90α and HSP90β levels in human specimens from 20 subjects with NAFLD (Table S1). HSP90β in the liver of NAFLD was higher compared with that in healthy donors, while HSP90α remained unchanged (Figure 1A). Serum total triglyceride (TG) and cholesterol (TC) concentration had high-positive correlation with HSP90β expression (*R*=0.4386, *P*<0.01;* R*=0.2066, *P*<0.05, respectively) (Figure 1B). No correlation between blood glucose or insulin and HSP90β was observed (Figure 1B). In contrast, there were no correlations between HSP90α level and these clinical parameters (Figure 1B). Next, the expression of these two HSP90s in diet induced obese mice and in *ob/ob* mice was checked. We observed that the hepatic mRNA and protein level of *HSP90AB1*, but not *HSP90AA1*, was largely increased in high fat diet (HFD)-fed mouse liver (Figure 1C-F), as well as in *ob/ob* mice (Figure 1G-J). All together, these results suggest that HSP90β, rather than HSP90α, is correlated with lipid dysregulation in NAFLD.

### Knockdown of HSP90β improves lipid homeostasis through inhibiting *de novo* lipid biogenesis

**Figure 2:**
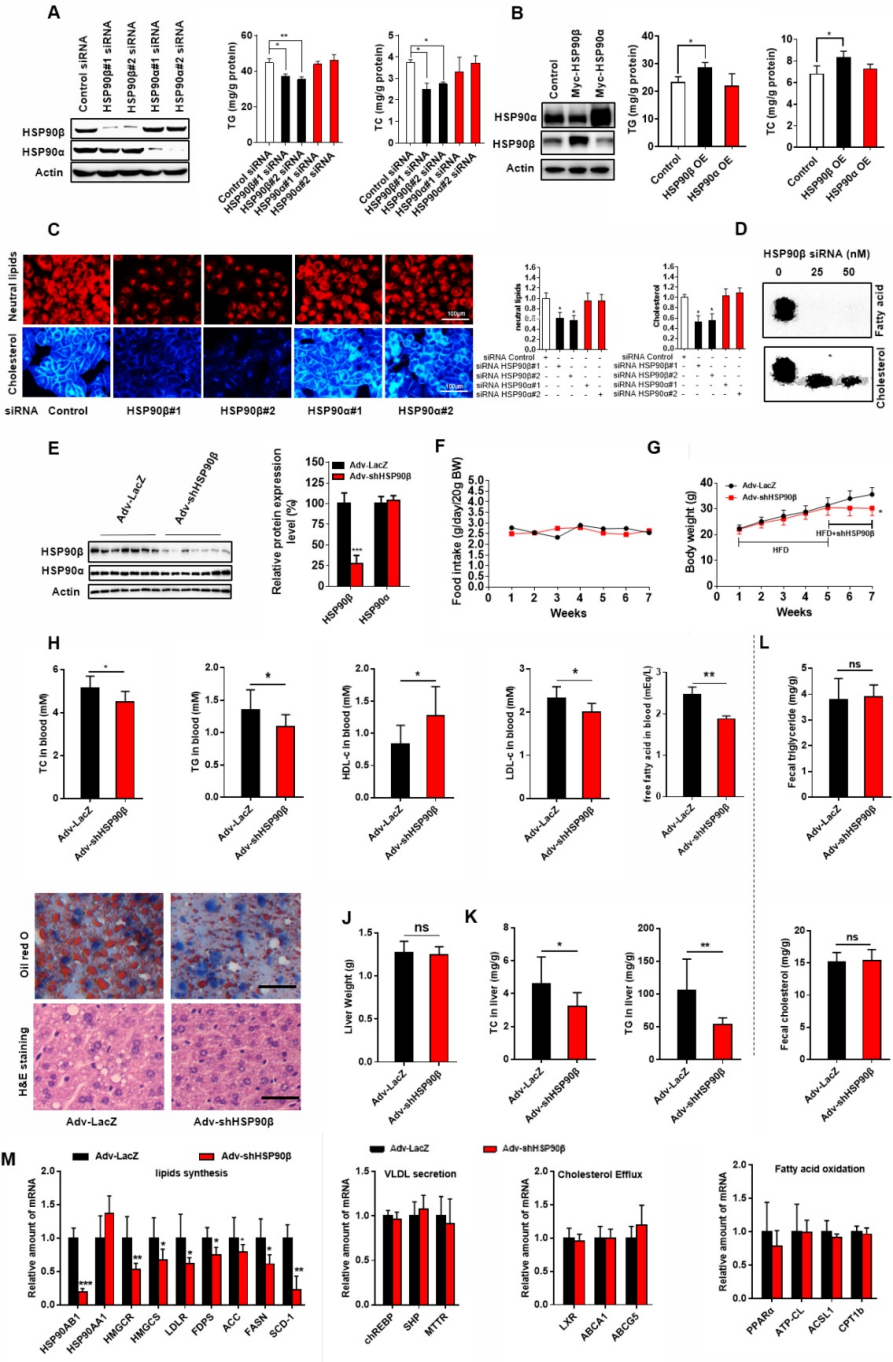
** Knockdown of HSP90β Improves Lipid Homeostasis.** (A and B) The cellular TG and TC levels were measured in human liver HL-7702 hepatocytes transfected with siRNA targeting HSP90β or HSP90α (A) or overexpression (OE) of HSP90β (3.6 times) or HSP90α (4.2 times) (B), the effect of interference or overexpression was verified by western blot. (C) The siRNA treated HL-7702 hepatocytes incubated in medium B for 48 h, and then switched to medium D for another 24 h. The cells then were stained with Nile-Red, which specifically recognizes neutral lipids or filipin, which definitely binds free cholesterol. (D) HL-7702 hepatocytes were incubated in medium B treating with indicated concentrations of siRNA HSP90β for 48 h, and then switched to medium D for another 24 h. Acetic acid sodium salt 1-^14^C was directly added into the medium and incubated for additional 2 h. The radioactive products were identified by comparison with unlabeled standards and visualized with iodine vapor. (E-L) Male C57BL/6J mice (6 weeks) were randomly grouped (10 mice each group). Mice were allowed *ad libitum* access to water and high fat diet (HFD). After four weeks, mice were intravenously injected with titer of 5×10^9^ adenovirus expressing the shRNA targeting HSP90β or the shRNA targeting LacZ. HFD was still administrated to mice for additional 14 days. Then, mice were sacrificed and subjected to various analysis. (E) The total protein from mice liver were prepared, and subjected to immunoblot analysis. Statistical analysis of hepatic HSP90β protein level in panel E normalized to Actin. (F) Food intake during the six week treatment. (G) Body weight measurement during the six weeks treatment. (H) The effect of HSP90β knockdown on serum TG, TC, HDL-c and LDL-c levels. (I) Oil red staining of liver sections. (J) The weight of liver. (K) The TC and TG level in the liver. (L) The TC and TG in the feces were analyzed by GC-MS. (M) Lipid mebabolism related genes expression was detected by q-RT-PCR. Mouse GAPDH was used as the internal control. Error bars are represented as mean ± SEM. Statistical analysis was done with one-way ANOVA (Dunnett's post test) (A, B and C), two-way ANOVA (Bonferroni's test) (F and G), or student's *t*-test (E, H, J, K, and L). *p < 0.05, **p < 0.01, ***p < 0.001 *vs* control vector, control adenovirus or control siRNA.

To check whether modulation of HSP90 paralogs indeed regulates lipid homeostasis, HL-7702 hepatocytes were transfected with HSP90 siRNAs (Figure 2A) or overexpression plasmids (Figure 2B). Knockdown of HSP90β reduced 21% of cellular TG and 33% of TC (Figure 2A). On the contrary, overexpression of HSP90β increased 24% of TG and 22% of TC (Figure 2B). By contrast, modulating HSP90α did not affect lipid contents (Figure 2A-B). Nile-Red and filipin staining gave the same results (Figure 2C). Moreover, knockdown of HSP90β significantly inhibited the *de novo* synthesis of both cholesterol and fatty acid in hepatic cells (Figure 2D).

To verify the *in vivo* role of HSP90β in regulating lipid homeostasis, adenoviruses carrying HSP90β-shRNA were injected into HFD-fed mice *via* the tail vein, leading to an 80% decrease of hepatic HSP90β (Figure 2E). Simultaneously, the expression of HSP90α was not affected (Figure 2E). In the absence of significant differences in dietary intake (Figure 2F), the body weight was about 16% lower than that in the LacZ-shRNA group, after injection of adv-shHSP90β for 2 weeks (Figure 2G). After HSP90β was knocked down, the levels of serum TC and TG were reduced by 13.2% and 19.0% respectively, but high-density lipoprotein cholesterol (HDL-c) increased about 46.1% (Figure 2H). The level of serum low-density lipoprotein cholesterol (LDL-c) was also decreased by 13.5% (Figure 2H). Albeit no significant difference in liver weight between the two groups (Figure 2J), a 30.0% decrease in hepatic TC and a 49.3% decrease of TG were observed (Figure 2K). Clearly, this change was not due to increasing fecal excretion of TC or TG (Figure 2L). Consistently, the size of hepatic lipid droplets dramatically decreased in HSP90β knockdown mice (Figure 2I). The expression of genes involved in the biosynthesis of cholesterol, fatty acid and triglycerides were lower in HSP90β knockdown mice (Figure 2M). While the expression of genes involved in VLDL secretion, cholesterol efflux and fatty acid oxidation was not significantly changed (Figure 2M). Therefore, we reasoned that the knockdown of HSP90β improves lipid homeostasis, most probably through inhibiting DNL.

HSP90 have a plethora of client proteins 22. For example, in adipocytes, HSP90 chaperones PPARγ to facilitate adipocytes survival and differentiation 4. Indeed, we observed a reduction of cell size in adipose tissues in adv-shHSP90β treated mice (Figure S1A). TNF-α and IL-6 expression did not change, suggesting there is no inflammation (Figure S1B). We did not observe changes of browning and lipid metabolism related genes in BAT and WAT (Figure S1B). It is possible that HSP90α and HSP90β may recognize and selective bind to different client proteins. Through Hsp90Int.DB website database query and alignment, we found that GZMA and H2AFX are specific client proteins for HSP90α, but not regulated by HSP90β 23, 24. Thus, only when HSP90α was knocked down, the levels of GZMA and H2AFX proteins were decreased (Figure S2). The functional difference of HSP90 paralogs might attribute to the difference of their client proteins.

### Corylin is a specific inhibitor of HSP90β

**Figure 4:**
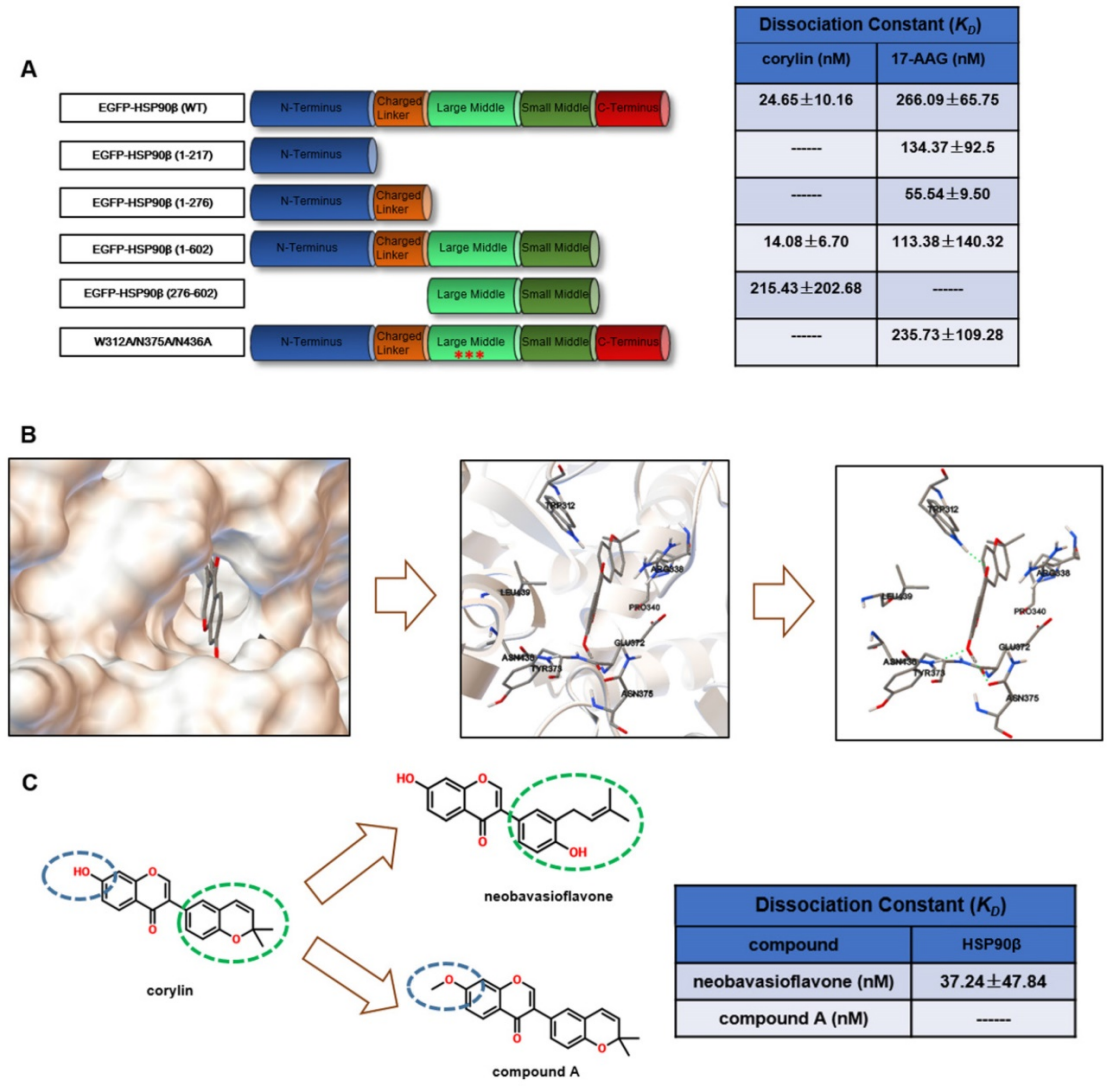
** Corylin Binds specifically to HSP90β.** (A) Various EGFP-HSP90β truncation mutant plasmids were constructed and the recombination mutant proteins of EGFP-HSP90β were purified. The interaction between different truncated or a triple mutant (W312A, N375A, N436A) form of HSP90β and 17-AAG or corylin was detected by microscale thermophoresis (MST). (B) Molecular docking model of corylin binding to HSP90β. Molecular modeling suggested that corylin binds to a pocket within MD composed of residues 312 to 439. Hydrogen bonds are formed between corylin and W312, N375, N436 of EGFP-HSP90β. (C) Schematic diagram of key structural difference among corylin, compound A and neobavaisoflavone. The interaction between recombination proteins EGFP-HSP90β and corylin, compound A or neobavaisoflavone was detected by microscale thermophoresis (MST).

The above results suggested that selectively inhibiting HSP90β may be a potential therapeutic strategy for lipid disorders. As the discovery of HSP90β specific inhibitors is not satisfying 29, we decided to use new tactics. We performed virtue screening to search compounds that have higher binding affinity to HSP90β (PDB ID code 3PRY) than to HSP90α (3Q6M). From a structure database of around 3000 in-house natural compound library, we found 12 compounds with the big difference in the binding energy between HSP90β and HSP90α. The effects on SREBP activity of these compounds were further evaluated (Table S2). It turned out that 9 out of 12 of the compounds exhibited SREBPs inhibitory effects, suggesting paralog specific inhibition of HSP90β might particularly affect SREBP activity. Among these compounds, corylin exhibited highest SREBP inhibitory effects (Table S2). Next, we used photo-affinity labelling and pull-down experiments to identify the interaction proteins to corylin 30. According to the experiment reproducibility (p<0.05) and the enrichment of proteins (>1.2 fold enrichment) by corylin-agarose beads, we finally focused on three proteins (HSP90β, HSP90α and ENO1) (Table S3). The binding affinity of corylin to these three proteins was then analyzed using microscale thermophoresis (MST) assay 31. The *K*_D_ between corylin and HSP90β was 24.65 ± 10.16 nM, much lower than that of 17-AAG (Figure 4A and Figure S4A-B). 17-AAG, but not corylin bound with HSP90α (Figure S4C-D). AP-III-a4 (a specific inhibitor of ENO1), but not corylin bound with ENO1 (Figure S4E-F). Collectively, the above results clearly indicate that the binding of corylin to HSP90β is specific. Consistently, the inhibitory effects of corylin on SREBPs activity were reversed by overexpression of HSP90β (Figure S5A-C), but not HSP90α or ENO1 (Figure S5D-E). 17-AAG binds to the NTD of HSP90, while, corylin did not bind to the NTD (1-217), the charged linker domain (218-276) and the C-terminal domain (CTD) (Figure 4A and Figure S6A-F). Residues between 276 and 602 are crucial for corylin binding (Figure 4A and Figure S6G-H). 17-AAG and XL-888, but not corylin, significantly inhibited the ATPase activity of HSP90β (Figure S7A), consistent with the binding data (Figure 4A). Next, we identified a single potential binding pocket located around residues 312 to 439 in the MD of HSP90β using Autodock (Figure 4B), a pocket that is distinguishable from HSP90α 32. The binding energy between HSP90β and corylin is -10.5 KJ/mol. In contrast, the binding of HSP90α and corylin is poor (-5.0 KJ/mol), which is similar to the results using Glide 6.9 (Table S2). More specifically, we found three hydrogen bonds formed between the amino acid residues of HSP90β MD and corylin based on surface topography and sequence of HSP90β. Two hydrogen bonds are predicted to form between the phenolic hydroxyl group and amino acids N375 and N436. Another hydrogen bond is formed between carbonyl group and W312 (Figure 4B). The residues W312, N375 and N436 were then mutated to alanine to prevent hydrogen bond formation, consequently, the mutated HSP90β no longer bound to corylin (Figure 4A and Figure S6I). By contrast, the point mutations did not influence the interaction between HSP90β and 17-AAG (Figure 4A and Figure S6J). Consistently, either mutation in these three amino acids, or corylin treatment, did not affect HSP90β ATPase activity (Figure S7B). When corylin was modified to neobavaisoflavone, the binding affinity was only slightly reduced (Figure 4C and Figure S6K). When phenolic hydroxyl group was converted to methoxy group (compound A, Figure 4C), the binding to HSP90β was significantly weakened (Figure 4C and Figure S6L). Consistently, the effect on SREBP activity of neobavaisoflavone was similar as corylin, while compound A no longer affected the abundance and transcriptional activity of SREBPs (Figure 4M and N). Thus, we concluded that the residues W312, N375 and N436 of HSP90β are essential for corylin binding.

### Corylin inhibits *de novo* lipid synthesis by promoting mSREBPs ubiquitination and proteasomal degradation

**Figure 5:**
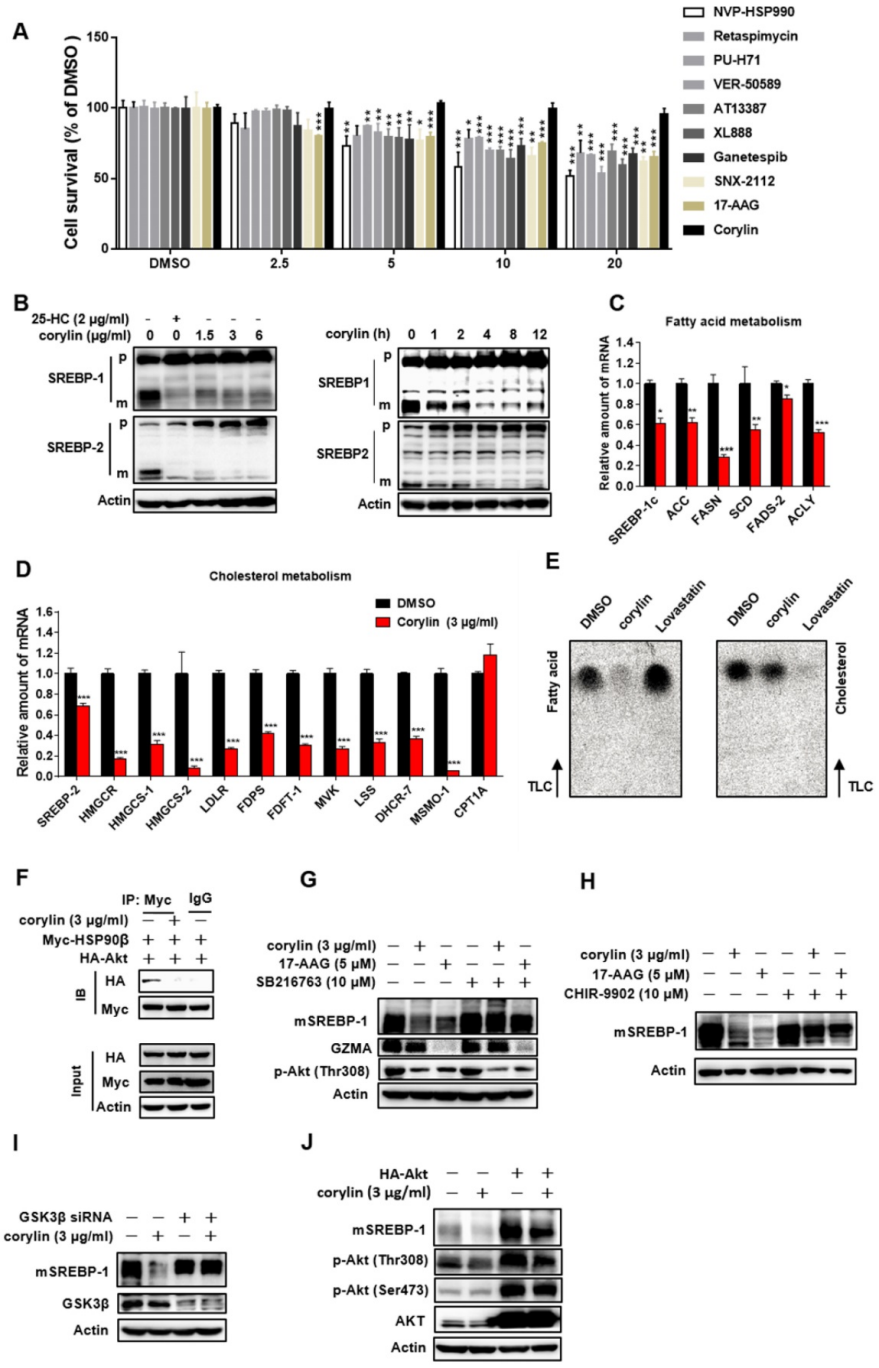
** Corylin Inhibits *de novo* Lipids Synthesis by Regulating SREBPs Activity.** (A) HL-7702 cells were treated with HSP90 inhibitors with different structure backbone. Only corylin did not cause cytotoxicity. (B) HL7702 cells were depleted of sterols by incubating in medium D for 24 h, and then switched to medium D containing indicated concentration of corylin for 4 h, or corylin (3 μg/ml) for different time, the whole cell extracts underwent immunoblotting with indicated antibodies. (C-D) HL7702 cells were depleted of sterols by incubating in medium D for 24 h, and then switched to medium D containing 3 µg/ml corylin for 4 h. The expression of various genes was analyzed by qRT-PCR. (E) HL7702 cells were depleted of sterols by incubating in medium D for 24 h, and then switched to medium D containing 3 μg/ml corylin or 2 μM lovastatin for 16 h. Acetic acid sodium salt 1-^14^C was directly added into the medium and incubated for additional 2 h. (F) 293T cells were transfected with Myc-HSP90β and HA-Akt for 24 h, after the treatment, the cells were incubated with medium D containing corylin for another 4 h. Cells were lysed and pulled down by Myc antibody. HL-7702 hepatocytes were treated with GSK3β inhibitor SB216763 (10 µM) (G) or CHIR-9902 (10 µM) (H) for 1 h, the cells were switched to medium D supplemented with inhibitors plus vehicle, or 3 µg/ml corylin for 4 h. The whole cell extracts underwent immunoblotting with indicated antibodies. (I) HL-7702 hepatocytes were transfected with GSK3β siRNA for 48 h, after the treatment, the cells were switched to medium D treated with 3 µg/ml corylin for 4 h. (J) HL-7702 hepatocytes were transfected with HA-Akt for 24 h, after the treatment, the cells were switched to medium D treated with 3 µg/ml corylin for 4 h. Whole cell extracts underwent immunoblotting with indicated antibodies. Error bars are represented as mean ± SEM. Statistical analyses were done with one-way ANOVA (Dunnett's post test) (A and C). *p < 0.05, **p < 0.01, ***p < 0.001 *vs* DMSO.

Corylin did not cause obvious hepatic toxicity (Figure 5A). In contrast, a series of pan-HSP90 inhibitors exhibited severe hepatic toxicity (Figure 5A). This is probably due to inhibition of HSP90α, since knockdown of HSP90α led to cell death (Figure S8A). Corylin treatment impaired the levels of mSREBP-1 and -2 in time- and dose- dependent manners (Figure 5B). Nevertheless, ATF6, a transcription factor with similar processing route as SREBPs, was not affected by corylin (Figure S8B), suggesting that the effect of corylin on SREBPs is specific. As corylin reduced the mSREBPs in the nucleus (Figure S8C), the expression of their target genes was significantly reduced (Figure 5C-D). These results were reproducible in human primary hepatocytes (Figure S9A-D). Consistently, DNL was significantly attenuated by corylin (Figure 5E). Furthermore, corylin largely reduced cellular cholesterol and neutral lipids level (Figure S8D).

In HSP90β knockout hepatocytes (KO cells), corylin no longer influenced mSREBPs (Figure S10A). KO cells got sensitive to corylin when wild-type HSP90β, but not triple mutant (W312A/N375A/N436A) HSP90β was put back (Figure S10B). Corylin decreased endogenous and exogenous mSREBPs (Figure S10C) through increased ubiquitination (Figure S10D) and accelerated degradation (Figure S10E and F). These effects were blocked by the proteasome inhibitor MG-132 (Figure S10G) or by FBW7 siRNAs (Figure S10H-I). Corylin disrupted the binding of HSP90β with Akt (Figure 5F) and significantly reduced Thr308 phosphorylation of Akt and Ser9 phosphorylation of GSK3β in a time- and dose-dependent manner (Figure S10K-L). Notably, in HSP90β KO cells, insulin no longer increased T308 phosphorylation, but still increased S473 phosphorylation. T308 dephosphorylation by corylin decreased ~55% of Akt activity, a much weaker effect than 17-AAG that inhibited ~80% of Akt activity (Figure S11). GSK3β S9 phosphorylation and mature form of SREBP-1c were downregulated in insulin treated HSP90β KO cells (Figure S10J). These data suggest that HSP90β is involved in insulin stimulated Akt activation, subsequently affect GSK3β-SREBPs and lipid biosynthesis in hepatocytes. Meanwhile, the decreased levels of mSREBPs was reversed by the GSK3β inhibitors SB216763 (Figure 5G), CHIR9902 (Figure 5H), or GSK3β siRNAs (Figure 5I and Figure S12A). Overexpression of Akt also blocked the effect of corylin on mSREBPs (Figure 5J and Figure S12B-D). Taken together, these data demonstrated that corylin, as a specific inhibitor of HSP90β, promotes mSREBPs ubiquitination and proteasomal degradation through the Akt-GSK3β-FWB7 pathway.

### Corylin improves lipid homeostasis and insulin resistance

**Figure 6:**
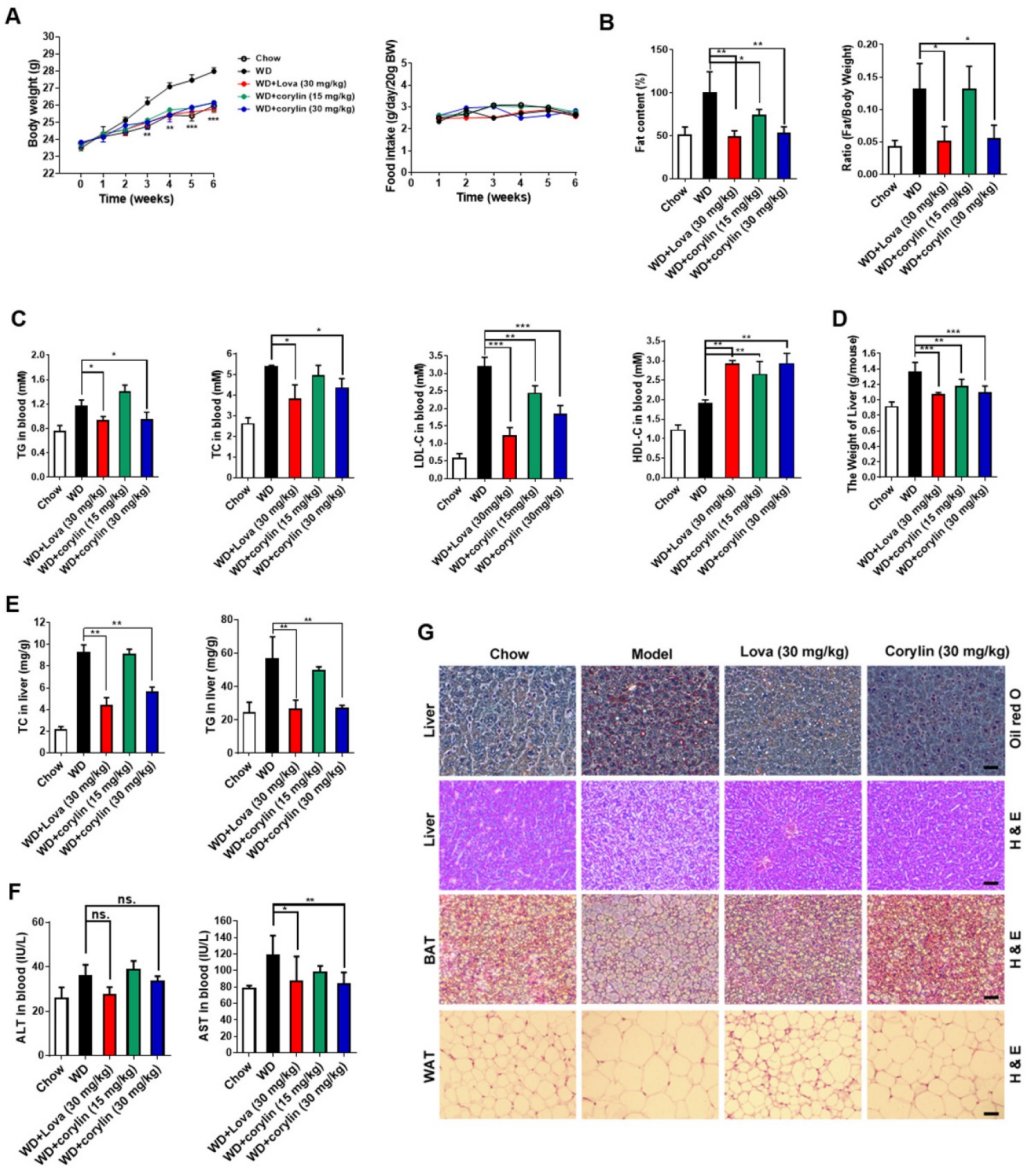
** Corylin Improves Lipid Homeostasis in WD-fed mice.** Male C57BL/6J mice at 6 weeks of age were randomly grouped (n=6). Mice were allowed *ad libitum* access to water and different types of diets (WD, western-type diet). Vehicle (0.5% CMC-Na), corylin (15 or 30 mg/kg), or lovastatin (30 mg/kg) was administrated to mice by gastric irrigation every day. After 6 weeks treatment, the mice were sacrificed and subjected to a series of analysis as indicated below. (A) Food intake and body weight. (B) The ratio of fat and body weight or lean. (C) Effect of corylin on serum TG, TC, LDL-c and HDL-c levels. (D) The weight of liver. (E) Effect of corylin on TG and TC levels in the liver. (F) Effect of corylin on Alanine aminotransferase (ALT) and aspartate transaminase (AST) in the serum. (G) Oil red staining in liver and histological analysis of liver, WAT and BAT. Error bars are represented as mean ± SEM. Statistical analyses were done with two-way ANOVA (Bonferroni's test) (A) or one-way ANOVA (Dunnett's post test) (B-G). *p < 0.05, **p < 0.01, ***p < 0.001 vs WD.

Next, we investigated the effect of corylin on obesity *in vivo*. The bodyweight of mice fed with western-type diet (WD) plus corylin (15 mg/kg/day and 30 mg/kg/day, respectively) or lovastatin (30 mg/kg/day) was lighter than that of vehicle treated WD mice while the food intake remained almost unchanged (Figure 6A). Meanwhile, the fat/lean or fat/whole-body ratio dropped more than 50% in the same dosage (30 mg/kg/day) of corylin- or lovastatin-treated mice (Figure 6B). Corylin or lovastatin significantly reduced the level of serum TC, TG and LDL-c, and increased the level of HDL-c (Figure 6C). The liver weight, hepatic TC and TG in corylin-treated mice were notably lower than those of the vehicle-treated mice (Figure 6D and E). Corylin significantly decreased the level of serum AST in WD-fed mice. No significant difference but reduced ALT level was detected in WD-fed mice with corylin (Figure 6F). Corylin at 30 mg/kg/day dramatically reduced the cell sizes of both white adipose tissue (WAT) and brown adipose tissue (BAT) in WD-fed mice. The contents of lipids were decreased in the livers of corylin-treated mice (Figure 6G). Similar body weight lowering effects were observed in HFD-fed mice (Figure S13A). Notably, there was no significant difference in food intake (Figure S13B), faecal cholesterol or triglyceride content (Figure S13C-D). Corylin treatment did not increase cold resistance in HFD-fed mice when exposed to cold (Figure S13E). The energy expenditure was then measured for 24 h under a 12 h light-day cycle. Interestingly, although no significant difference was observed at night between the HFD-fed mice and HFD plus corylin-fed mice, the O_2_ consumption and energy expenditure (EE) were significantly increased during the day (Figure S13F-H). The EE increase also contributed, at least partially, to the body weight loss in HFD-fed mice. Weight reduction is considered to be beneficial for improving insulin resistance. Next, we investigated whether corylin improves insulin resistance *in vivo*. Corylin significantly blunted the elevated fasting blood glucose and insulin level (Figure S14A-B) and improves the glucose and insulin intolerance (Figure S14C-D) in WD-fed mice. Consistent with the *in vitro* results, corylin reduced the level of mSREBPs in the liver of WD-fed mice (Figure S14E-F). Most of SREBP-1 and SREBP-2 target genes were significantly downregulated in the liver of WD-fed mice by corylin (Figure S14G-I). In hepatocytes, as well as in mice livers, corylin neither increases LXR target gene expression, nor activates ER stress genes expression (Figure S15). We also found that the expression of VLDL secretion, cholesterol efflux, fatty acid oxidation, adipocyte browning, and glucose uptake were not significantly changed (Figure S16).

To address the question whether corylin improves liver metabolism through inhibiting HSP90β, we treated HFD-fed mice with corylin for 4 weeks, then adenoviral virus expressing HSP90β or control were injected. Overexpression of HSP90β reversed the corylin effects on SREBPs and their target gene expression (Figure S17A-H). HSP90β also reversed lipid parameters that were improved by corylin (Figure S17I-M). In summary, corylin inhibits SREBPs and improves lipid homeostasis *in vivo* through inhibiting HSP90β.

### Corylin decreases atherosclerosis development

**Figure 7:**
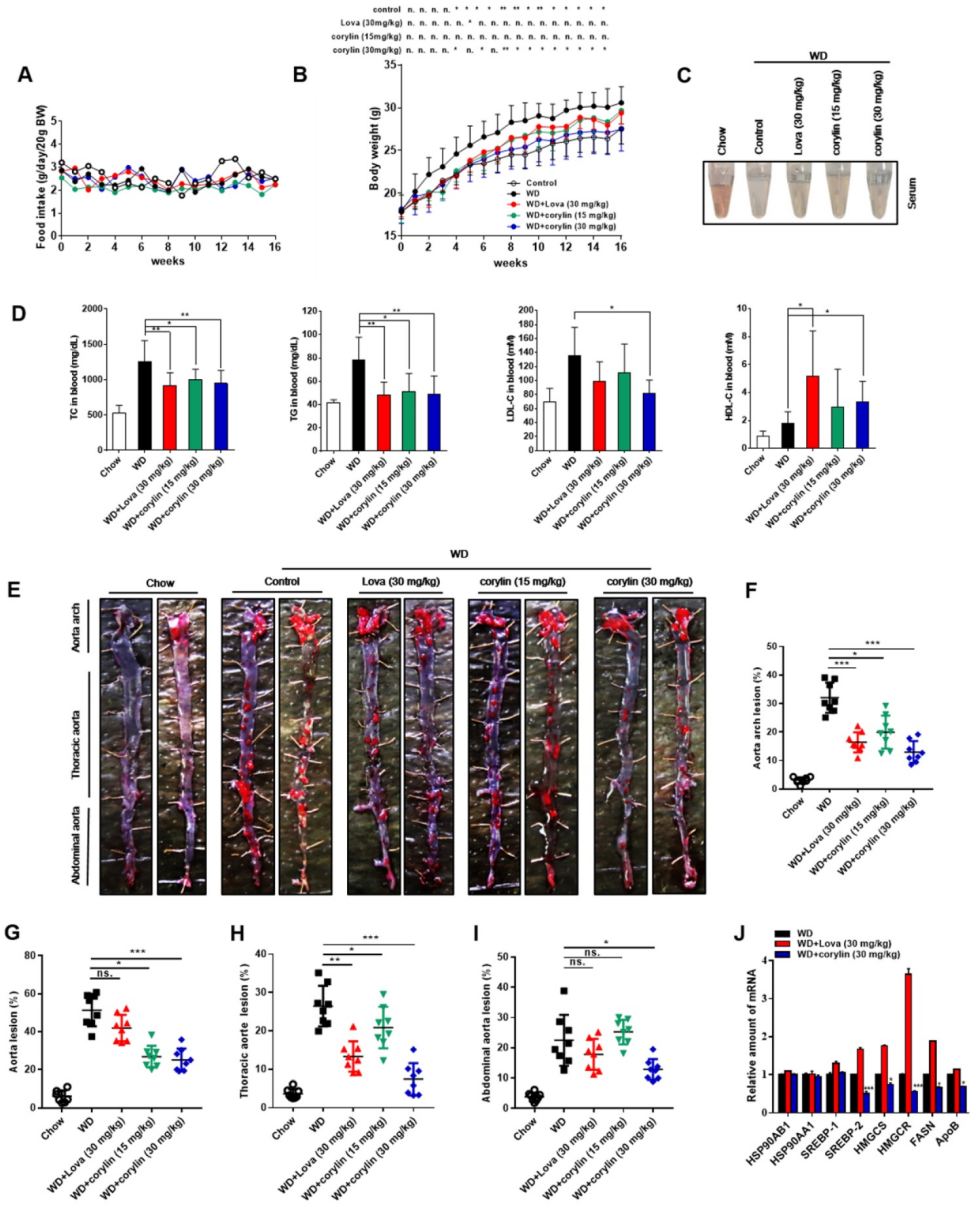
** Corylin Decreases the Atherosclerosis Development in ApoE^-/-^ Mice.** Six-week-old male ApoE^-/-^ mice were randomly grouped (n=12) and fed with WD supplemented with vehicle (0.5% CMC-Na), corylin (30mg/kg) or lovastatin (30 mg/kg) for 16 weeks. After the treatment, the mice were sacrificed and subjected to various analyses as described below. (A) Food intake during the 16 weeks experiment period. (B) Body weight of each group during the 16 weeks experiment period. (C) Representative photographs of plasma from each group. (D) The levels of TC, TG, LDL-c and HDL-c in plasma after 16 weeks treatment. (E) Representative photographs from en face analysis of aortas from different groups after 16 weeks treatment. (F-I) Quantification of the lesion areas in (E). (J) qRT-PCR analysis of gene expression in ApoE^-/-^ mice liver after 16 weeks. For each group, equal amounts of total RNA from three mice were subjected to qRT-PCR quantification. Error bars are represented as mean±SEM. Statistical analyses were done with two-way ANOVA (Bonferroni's test) (A and B) or one-way ANOVA (Dunnett's post test) (D, and F-J). *p < 0.05, **p < 0.01, ***p < 0.001 vs WD.

The effect of corylin on atherosclerotic lesion formation was further explored in ApoE^-/-^ mice. Corylin treatment did not cause obvious toxicity and difference in food intake (Figure 7A), but reduced the weight gain at high dose (Figure 7B). Both corylin and lovastatin treatment made the plasma transparent, indicating reduced lipid content (Figure 7C). Moreover, corylin or lovastatin obviously decreased TC, TG and LDL-c levels and elevate HDL-c levels in plasma (Figure 7D). Corylin reduced the number and size of aortic plaques in WD-fed ApoE^-/-^ (Figure 7E and F). Particularly, the areas of the lesion in the thoracic aorta and abdominal aorta were significantly smaller (Figure 7G-I). The lesion areas in cross-sections of the aortic sinus in the corylin-treated mice were reduced dramatically (Figure S18A). The activated endothelial cells marker VCAM-1 was reduced in corylin-treated mice (Figure S18B). Upon corylin treatment, the expression of *HSP90AB1* and *HSP90AA1* remained unchanged, while *SREBPs* and their target genes involved in lipid synthesis were downregulated (Figure 7J). In contrast, *HMGCR*, *HMGCS* and *FASN* were dramatically upregulated by lovastatin (Figure 7J). These results demonstrated that corylin ameliorates atherosclerosis lesion formation of plaques in the aortic root in WD-fed ApoE^-/-^ mice without increasing fatty acid synthesis, a feature superior to lovastatin.

## Discussion

In this report, we have demonstrated the clinical significance of the expression of HSP90β in NAFLD patients. Further, a novel paralog-specific HSP90 inhibitor, corylin has been identified. Genetic targeting or pharmacologic inhibition of HSP90β leads to degradation of SREBPs *via* Akt-GSK3β-FBW7 pathway, thus improves lipid homeostasis.

To date, the paralog-specific role of HSP90 in the liver is little known. In this study, we observed HSP90β, instead of HSP90α, obviously increases in the liver of human NAFLD patients and obesity- induced fatty liver mice (Figure 1). Further studies indicate that HSP90β plays a paralog-specific role in regulating lipid homeostasis. Our results help researchers to understand the role of different paralogs of HSP90 in lipid metabolism, and promote HSP90β as a drug target for the treatment of lipid disorders. Chemical compounds that specifically inhibit HSP90β certainly satisfy the demand of precise medical treatment for lipid disorders. The NTD with ATP pocket- and CTD with novobiocin-binding site of HSP90 are under intense study and therefore attract most interest in drug discovery. Small compounds such as 17-allylaminogeldamamycin (17-AAG), ganetespib (STA-9090), SNX-2112 and novobiocin have been developed accordingly 33. The NTD is not only homology to HSP90 chaperone family members, but also to members of the gyrase-Hsp90-histidine kinase-MutL (GHKL) group within the ATPase/ kinase superfamily 34. Similarly, the CTD contains a second ATP binding site, which can be competed with novobiocin, cisplatin, epilgallocatechin-3-gallate (EGCG) and taxol 35. These two binding domains are closely related to cell proliferation and tumor progression 36. Similar as other drugs targeting ATP/ADP binding pocket, the off-target effects hampered the development of the first generation geldanamycins that exerted significant toxicities 37, 38. Indeed, we observed cytotoxicity of these compounds in hepatocytes (Figure 5A). Corylin binds to HSP90β in a unique pocket located in the middle domain formed by amino acids W312/N375/N436, notably, either mutation in these three amino acids, or corylin treatment, did not affect HSP90β ATPase activity (Figure S7B). The *K_D_* of corylin with HSP90β was 24.65 ± 10.16 nM, the highest binding affinity ever reported. Therefore, corylin is the small molecule that distinguishes HSP90α and HSP90β. With this paralog-specific inhibitor, less cytotoxicity was observed (Figure 5A) and further understanding of the biologic function of this chaperone family is warranted.

In this study, we found genetic deletion or pharmacological inhibition of HSP90β caused the Thr308 dephosphorylation of Akt (Figure S2F, Figure S10J-L). Akt full activation needs both T308 in the catalytic domain and S473 in the carboxy-terminal noncatalytic region 39. It is reported that either T308 or S473 mutation leads to diminished Akt activity, although T308 mutation seems to cause more severe effect 40. The downstream Akt targets are affected differently by Akt mutations. For example, it has been shown that genetic ablation of mTORC2 components (rictor, mLST8 or sin1) abolished S473 phosphorylation and only affected a subset of Akt targets *in vivo*, including FoxO1/3a, while other Akt targets, TSC2, GSK3, S6K and 4E-BP1 were unaffected 41. As in our case, we found that genetic or pharmacologic inhibition of HSP90β caused T308 dephosphorylation. T308 dephosphorylation by corylin decreased ~55% of Akt activity, a much weaker effect than 17-AAG that inhibited ~80 % of Akt activity (Figure S16). Subsequently, GSK3β was then activated and stimulated SREBP degradation (Figure S2F and Figure S10). The effect could rather be a result of changes in overall Akt activity change than S473 phosphorylation, since T308 and S473 phosphorylation could be separated regulated 41. As corylin decreased T308 without affecting S473 very much, it could be a good tool to study mTORC2 independent Akt regulation.

Previous reports used pan-HSP90 inhibitor 17-AAG or its analogues to study the function role of HSP90 in different disease models 42. As 17-AAG binds both HSP90α and HSP90β, meanwhile, there is no HSP90β-flox mice available (HSP90β KO mice are lethal), the HSP90β specific role in physiological and pathological conditions remain largely unknown. With HSP90β specific inhibitor corylin, HSP90β specific roles in these diseases could be re-examined. It should also be noted that although alcoholic fatty liver disease (AFLD) and NAFLD share similar pathological spectra, these two diseases differ from each other not only in terms of clinical features and patient outcomes 43, but also in terms of underlying mechanisms 44. Notably, chemicals that protects against ethanol induced liver injury, such as silymarin 45, not necessarily alleviated NAFLD and vice versa 46. Whether inhibition of HSP90β in AFLD models also decreased alcohol mediated oxidative stress, reduced serum endotoxin, decreased inflammatory responses in hepatic macrophages, deserve further study.

In the last decades, the lab of Brown and Goldstein has discovered that SREBPs are master regulators of *de novo* lipogenesis 18, 47. These studies suggest that selective inhibition of SREBPs might constitute a new class of drugs against lipid disorders, as well as type 2 diabetes. However, the inhibitors of SREBPs processing, such as 25-HC, activate LXR and induce ER stress, leading to hepatic steatosis and insulin resistance 48, 49. Corylin suppressed SREBPs activity in a different mechanism. It selectively inhibits HSP90β and promotes SREBPs degradation depending on Akt-GSK3β-FBW7 pathway (Figure 4 and Figure S2 and Figure S10), without affect LXR target genes and ER stress gene expression. In animal experiments, corylin alleviates hepatic steatosis and hyperglycemia (Figure 6G), insulin resistance (Figure S14A-D). These data suggested that corylin has an incomparable advantage in the treatment of lipid disorders. In a previous study 50, it shows that corylin was subjected to massive first-pass metabolism in liver and intestine. Therefore, we supposed that liver is the major organ that responsible for the beneficial effects of corylin.

We believe our findings pave the way for future investigation on the paralog-specific roles of HSP90α and HSP90β in different physiological and pathological conditions. In the obese animals, both mRNA and protein level of *HSP90AB1* is increased. Therefore, unraveling the different molecular mechanisms that activate HSP90 paralogs is very important to advance the understanding of HSP90 functions, thus to improve the therapeutic effects in metabolic disease, cancer, neurodegeneration diseases, eplipsy, etc.

## Supplementary Material

Supplementary figures and tables.

## Figures and Tables

**Figure 3:**
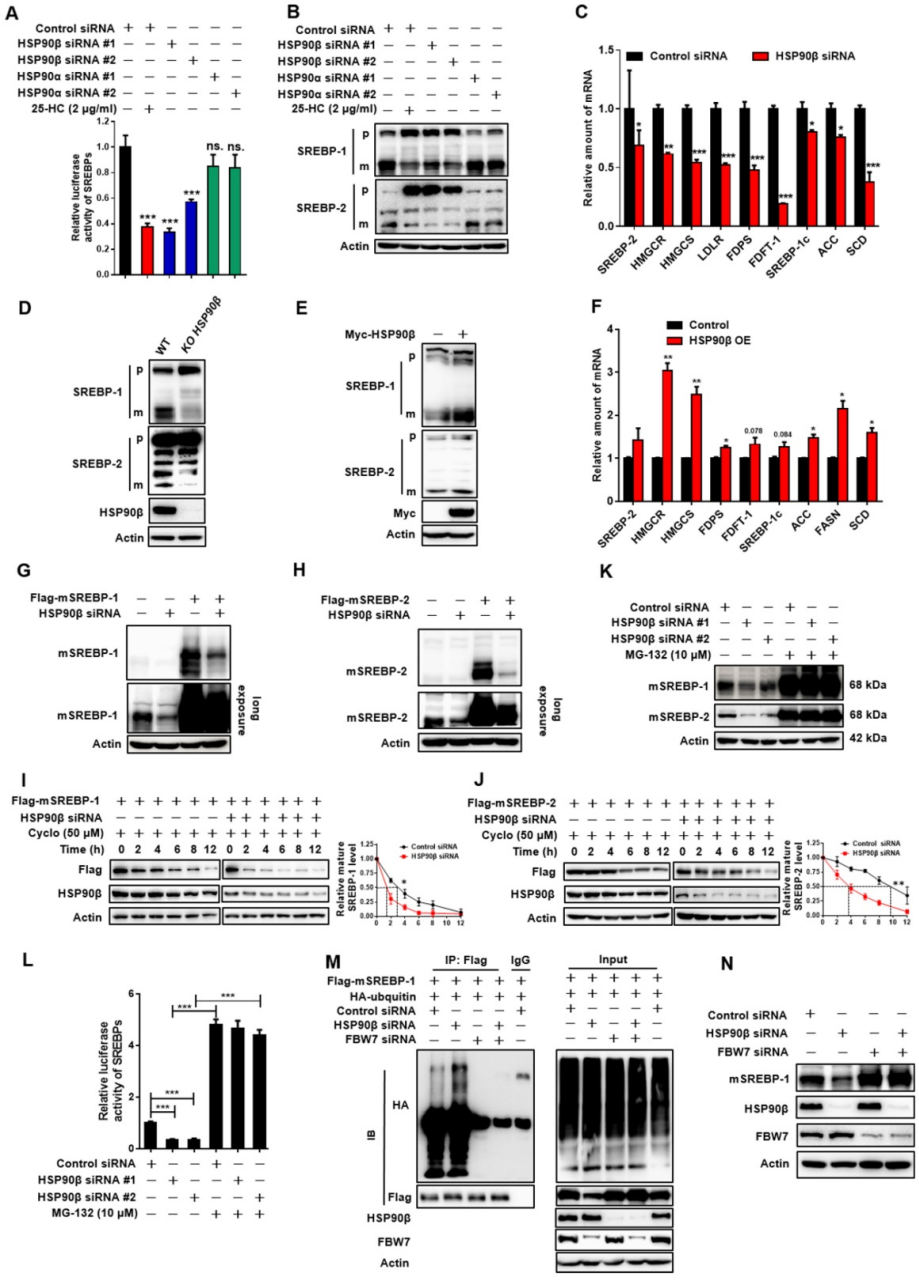
** HSP90β Regulates *de novo* Lipid Synthesis through Promoting the mSREBPs Ubiquitination and Proteasomal degradation.** (A) HL-7702/SRE-Luc cells were incubated in medium B and treated with indicated siRNA for 48 h, the cells were then switched to medium D or medium D containing 25-HC (2 μg/ml) for 4 h. After the treatment, cells were lysed and luciferase activity was measured. (B and C) HL-7702 hepatocytes were incubated in medium B and treated with indicated siRNA for 48 h, the cells were then switched to medium D or medium D containing 25-HC (2 μg/ml) for 4 h. After the treatment, (B) the whole cell extracts underwent immunoblotting with indicated antibodies. P represents precursor SREBP and m represents mSREBP. (C) The expression of various genes was analyzed by qRT-PCR. Human GAPDH was used as the control. (D) The HL-7702/WT and HL-7702/KO HSP90β cells were depleted of sterols by incubating in medium D for 24 h, the total protein of cells were prepared and subjected to immunoblotting with indicated antibodies. (E and F) HL-7702 hepatocytes were transfected with myc-HSP90β (3.4 times) for 24 h, and then depleted of sterols by incubating in medium D for another 24 h, (E) the whole cell extracts underwent immunoblotting with indicated antibodies. (F) The expression of various genes was analyzed by qRT-PCR. Human GAPDH was used as the internal control. (G and H) HL-7702 hepatocytes were transfected with flag-mSREBP-1 (G) or flag-mSREBP-2 (H) plasmids and cultured for 24 h. The cells were switched to medium D for another 24 h, the whole cell extracts underwent immunoblotting with indicated antibodies. (I and J) HL-7702 hepatocytes were transfected with flag-mSREBP-1 plasmid and siRNA against HSP90β for 48 h (I). HL-7702 hepatocytes were transfected with flag-mSREBP-2 plasmid and siRNA HSP90β for 48 h (J). After adding 50 µM cycloheximide for 1 h, the whole cell extracts were harvested after incubation for indicated periods of time, mSREBPs were detected by immunoblotting with indicated antibodies. The graph depicted the averaged ratio of the autoradiographic signals of flag to actin levels, the dotted lines show the half-life of flag-mSREBP-1/-2. (K) HL-7702 hepatocytes were transfected with indicated siRNA for 48 h. The cells were switched to medium D containing MG-132 for 12 h, the whole cell extracts underwent immunoblotting with indicated antibodies. (L) HL-7702/SRE-Luc cells were transfected with HSP90β siRNAs for 48 h, luciferase activity was then measured. (M) 293T cells were transfected with flag-mSREBP-1 and HA-ubiquitin and with or without siRNA HSP90β and FBW7, ubiquitylated SREBP-1 was detected by immunoblot. (N) HL-7702 hepatocytes were transfected with the indicated siRNA for 48 h, the whole cell extracts underwent immunoblotting with indicated antibodies. Error bars are represented as mean ± SEM. Statistical analyses were done with one-way ANOVA (Dunnett's post test) (A, C, and E). *p < 0.05, **p < 0.01, ***p < 0.001 *vs* control siRNA or control vector.

## Abbreviations

ABCA1: ATP-binding cassette transporter 1
ACC: acetyl-CoA carboxylase
ACO: acyl-CoA oxidase
Akt: protein kinase B
ApoB: apolipoprotein B
ApoE: apolipoprotein E
BAT: brown adipose tissue
CPT1: carntine palmitoyltransferase 1
CYP7A1: cholesterol α-hydroxylase
Dio2: type 2 iodothyronine deiodinase
FASN: fatty acid synthase
FBW7: F-Box and WD40 Domain Protein-7
FDPS: farnesyl diphosphate synthase
Glut1: glucose transporter 1
Glut4: glucose transporter 4
GSK3β: glycogen synthase kinase 3 beta
HDL-c: serum high-density cholesterol
HFD: high fat diet
HMGCR: 3-hydroxy-3- methylglutary-CoA reductase
HMGCS: 3-hydroxy-3- methylglutary-CoA synthase
HL: hepatic lipase
HSP90AB1: heat shock protein 90 alpha family class B member 1
HSP90AA1: heat shock protein 90 alpha family class A member 1
Insig: insulin induced gene
LDL-c: serum low-density cholesterol
LDLR: low density lipoprotein receptor
LPDS: lipoprotein deficient serum
LPL: lipoprotein lipase
NAFLD: non-alcoholic fatty liver disease
PGC-1α: PPARγ- coactivator-1α
PPARγ: peroxisome proliferator-a ctivated receptor γ
RQ: respiratory quotient
SCAP: SREBP cleavage activated protein
SCD: stearoyl-CoA desaturase
SREBPs: sterol regulatory element- binding proteins
SR-BI: scavenger receptor class B member 1
TC: total cholesterol
TG: triglyceride
TNF-α: tumor necrosis factor α
UCP-1: uncoupling protein 1
UCP-2: uncoupling protein 2
