# Supplementary material for: Inhibition of HSP90β Improves Lipid Disorders by Promoting Mature SREBPs Degradation via the Ubiquitin-proteasome System
Source: Theranostics. 2019 Aug 12;9(20):5769–83. doi: 10.7150/thno.36505 (PMC6735373; doi:10.7150/thno.36505)
Supplement: Supplementary file 1 — Supplementary figures and tables. [file thnov09p5769s1.pdf]

Title: Inhibition of HSP90β Improves Lipid Homeostasis Disorder by Promoting Mature SREBPs Ubiquitin-proteasome Degradation

Materials and Methods

Materials

Corylin and neobavaisoflavone were purchased from shanghai u-sea bio-tech co., Ltd. (Shanghai, China). Compound A was synthesized by Prof. Hongbin Sun Lab (China Pharmaceutical University). 3-(4,5-dimethylthiazol-2-yl)-2,5-diphenyltetrazolium-bromide (MTT), cholesterol, 25-hydroxycholesterol (25-HC), insulin, mevalonate, Nile-Red and filipin were purchased from Sigma-Aldrich (St. Louis, MO, USA). Luciferase assay reagent and reporter lysis buffer from Promega (Madison, WI，USA). CHIR-9902, cycloheximide, MG-132, SB216763, AP-III-a4, SNX-2112, Retaspimycin, VER-50589, XL888, PU-H71, AT13387, NVP-HSP990, and Ganetespib were from MedChem Express (Shanghai, China). SB216763 was purchased from Selleckchem (Shanghai, China). Acetic acid sodium salt 1-^{14}C was from Perkin Elmer (Waltham, MA, USA). Lovastatin and compactin were purchased from Aladdin (Los Angeles, CA, USA). Fetal bovine serum (FBS), DMEM and F12K medium were purchased from GIBCO (New York, USA). Hepatocyte Medium HM (Cat. No. 5201) and Human Hepatocyte HH (Cat. No. 5200) were purchased from ScienCell Research Laboratories (San Diego, CA, USA). Protein A/G PLUS-Agarose immunoprecipition reagent (Cat. No.sc-2003) was purchased from Santa Cruz (Dallas, TX, USA). Lipoprotein-deficient serum (LPDS) was purchased from Kalen

Biomedical (Montgomery Village, MD, USA).

Primary antibodies

Anti-SREBP-1 (catalogue No. sc-8984) and anti-Myc (catalogue No. sc-40) antibodies were from Santa Cruz (Santa Cruz, CA, USA). Anti-HSP90β (catalogue No. ab203085), anti-phospho-GSK3β (Ser 9) (catalogue No. ab75814), anti-VCAM-1 (catalogue No. ab134047) and anti-FBW7 (catalogue No. ab179961) antibodies were purchased from Abcam (Cambridge, UK). Anti-Akt (catalogue No.4691), anti-phospho-Akt (Thr308) (catalogue No.9275), anti-phospho-Akt (Ser473) (catalogue No.4060), anti-flag-tag (catalogue No. 14793) and anti-ATF-6 (catalogue No.65880) antibodies were purchased from Cell Signaling Technology (Beverly, MA, USA). Anti-Histone H3 (catalogue No. A2348), anti-HSP90α (catalogue No. A0365), anti-HA-tag (catalogue No. AE008), anti-His-tag (catalogue No. AH367), anti-GZMA (catalogue No. A6231), anti-H2AFX (catalogue No. A11361) and anti-GSK3β (catalogue No. A11578) antibodies were purchased from ABclonal Technology (Wuhan, Hubei, China). Anti-SREBP-2 (catalogue No. NBP1-54446SS) antibody was from Novus Bio (Littleton, CO, USA). Anti-Actin (catalogue No. AF0003) antibody was purchased from Beyotime Biotechnology (Haimen, Jiangsu, China).

siRNAs

HSP90β#1 (GGAGAUUUUCCUUCGGGAGTT), HSP90β#2 (GGCUGAGG CCGACAAGAAUTT), HSP90α#1 (AGCGUUCAUGGAAGCUUUGTT), HSP90α#2 (GGAAAGAGCUGCAUAUUAATT), FBW7#1 (CCUUAUAUGGGCAUACUUCTT), FBW7#2

(GCACAGAAUUGAUACUAACTT) and GSK3β (CUCAAGAACUGUCAAGUAATT) were purchased from GenePharma (Shanghai, China). Scrambled siRNA (UUCUCCGAACGUGUCACGUTT) was used as control.

Plasmids

The pCMV3-HSP90β-Myc (catalog No. HG11381-CM), pCMV3-HSP90α-Myc (catalog No. HG11445-CM), pCMV3-HSP90β (W312A/N375A/N436A)-Myc, pCMV3-ENO1-His (catalog No. HG11554-NH) and pCMV3-FBW7-His (catalog No. HG13414-CH) expression plasmids were purchased from Sino Biological Inc. (Beijing, China). The pcDNA3.1-2×flag-mSREBP-1 and pcDNA3.1-2×flag-mSREBP-2 were gifts from Timothy Osborne (Addgene plasmid # 26802, and # 26807). The HA-ubiquitin expression plasmids were gifts from Prof. Hui Zheng (Soochow University). The HA-Akt expression plasmids were kindly provided by Prof. Yong Liao (Chong Qing Medical University). The pET28a-HSP90α-Myc, pET28a-HSP90β-Myc, pET28a-HSP90β(1-218)-Myc, pET28a-HSP90β(1-276)-Myc, pET28a-HSP90β(1-602)-Myc, pET28a-HSP90β(276-602)-Myc, pET28a-HSP90β(W312A/N375A/N436A)-Myc expression plasmids were constructed.

Primers

Supplementary Table 4

Culture medium

Medium A: DMEM containing 100 units/ml penicillin and 100 μg/ml streptomycin sulfate; Medium B: medium A supplemented with 10% FBS; Medium C: a 1:1

mixture of Ham's F-12 medium and DMEM containing 100 units/ml penicillin and 100 μg/ml streptomycin sulfate; Medium D: medium C supplemented with 5% LPDS, 10 μM compactin and 50 μM sodium mevalonate; Medium E: Hepatocyte Medium (HM) consists of 500 ml of basal medium, 25 ml of fetal bovine serum (FBS, Cat. No. 0025), 5 ml of hepatocyte growth supplement (HGS, Cat. No. 5252) and 5 ml of penicillin/streptomycin solution (P/S, Cat. No. 0503).

Viability assay

Viability assay was detected by MTT assay. Briefly, HL-7702 and HH cells were seeded at the density of 2×10^4 cells/well in 96-well plate and maintained under 5% CO_2 at 37 °C. After the treatment, cells were treated with corylin as indicated. After 24 h, 10 μl of MTT (5 mg/ml) was added and incubated for 4 h. The cytotoxicity of corylin was determined by microplate reader (Multiskan FC).

Measurement of *de novo* fatty acid and cholesterol synthesis

After the cells were treated, acetic acid sodium salt 1-^{14}C (20 μCi/100 mm^2 dish) was directly added and incubated for additional 2 h. The cells were washed and dissolved with 0.1 N NaOH, and cell suspensions were autoclaved for alkaline saponification. Then cholesterol were extracted in petroleum ether and evaporated to dryness with N_2. After addition of 12 N HCl, fatty acids were extracted in petroleum ether and evaporated to dryness. The lipids were resolved by thin-layer chromatography (Silica gel 60, Merck). The radioactive products were identified by comparison with unlabeled standards and visualized with iodine vapor.

qRT-PCR

Total RNA was extracted from HL-7702, HH cells or mice livers using Trizol reagent (Invitrogen) according to the manufacturer's instructions. RNA concentrations were equalized and converted to cDNA using a kit (Hiscript II reverse transcriptase, Vazyme). Gene expression was measured by qRT-PCR (Roche) using SYBR-green (Hiscript II reverse transcriptase, Vazyme). Expression was normalized to GAPDH. The sequences of primers used in the experiments were listed in Supplementary Table 4.

Western blot analysis

For whole cell lysate, cells were harvested and suspended in 150 μl of RIPA buffer (Beyotime, China) containing protease inhibitors (10 g/ml leupeptin, 5 g/ml pepstatin A, 25 g/ml ALLN, 1 mM PMSF). Protein concentration was determined according to BCA (Beyotime, China), then the extracts were mixed with 5×SDS loading buffer (Beyotime, China). After the mixtures were boiled at 95 °C for 10 min, they were subjected to SDS-PAGE, transferred to nitrocellulose membranes, and subjected to immunoblot analysis.

Nuclear extract was extracted by NE-PER nuclear and cytoplasmic extraction kit (Thermo). The pellets from 100-mm dishes of HL-7702 cells were harvested by trypsinization and collected by centrifugation at 500 g for 5 min and washed with PBS. Cells were transferred to 1.5 ml microcentrifuge tubes followed by centrifugation at 500 g for 3 min. After that, the supernatant layer was carefully removed by using pipette and discarded. The remaining cell pellet at the bottom was allowed to dry. Then cells were suspended in 500 μl of buffer A (10 mM HEPES, KOH (pH 7.6), 1.5

mM $MgCl_2$, 10 mM KCl, 5 mM EDTA, 5 mM EGTA, 250 mM Sucrose) containing protease inhibitors as described above, and then the tubes were vortex at the highest speed for 15 s to suspend the cell pellet and were put on ice and incubated for 10 min. Followed by the addition of ice-cold CER-II to the tubes and vortex again for 5 s and incubated again on ice for 1 min. Then, the tubes were vortex again and centrifuged at 13,000 g for 5 min. After centrifugation, the upper supernatant layer containing cytoplasmic extract was transferred to pre-chilled tubes. Cold NER was added to the remaining insoluble pellet, which contained nuclei. After NER was added, the tubes were vortex at the highest speed for 5 s and kept on ice for 10 min, this was repeated for three times. When the incubation finished, the tubes were centrifuged at 13,000 g for 10 min. The supernatant layer containing the nuclear extract was transferred to pre-chilled tubes. The next operation is the same as above.

Immunoprecipitation

Briefly, for co-immunoprecipitation assay, 293T cells were transfected with different plasmids (Myc–HSP90β, flag-mSREBP-1, flag-mSREBP-2, HA-ubiquitin, His-FBW7 and HA-Akt) for 24 h. Cells were washed three times and then lysed with lysis buffer (20 mM Tris-HCl, pH 7.5, 137 mM NaCl, 5 mM EDTA, 1% NP-40, 10% glycerol, 50 mM NaF, 1 mM Na_3VO_4 and protease inhibitor PMSF) for 30 min at 4 °C. The cell lysate were centrifuged for 10 min at 12,000 rpm at 4 °C. About 10% of the supernatant was used for western blot as inputs, while the rest of homogenates were incubated with indicated antibodies (Myc, HSP90β and IgG) overnight at 4 °C. Protein A/G plus agarose beads (Santa Cruz) were added at 4 °C for another 2 h. The

immunoprecipitation beads were washed with cold PBS for five times, followed by western blotting analysis.

Knockout of HSP90β by CRISPR-Cas9

Targeting sequences were designed at CRISPR direct (http://crispr.mit.edu), provided by the Zhang Lab, MIT (Massachusetts Institute of Technology) 2015. The knockout HSP90β HL-7702 cell line was generated by CRISPR-Cas9 targeting the following site: 5'- CACCGCCTGACAGACCCTTCGAAGT-3'. Briefly, HSP90β complementary oligonucleotides with BbsI restriction site for guide RNAs (gRNAs) were synthesized and cloned into pHBcas9/gRNA puro vector (HANBIO, HBCR-001) and confirmed by sequencing, named pHBcas9/HSP90β/gRNApuro. HL-7702 cells were transfected with pHBcas9/HSP90β/gRNApuro plasmid using Lipofectamine 2000 according to the manufacturer's instruction. After transfection for 48 h, cells were selected for 3 days in the presence of 2 μg/ml puromycin (Sigma, P7255). Single colonies were isolated and the gene knockout clones were confirmed by immunoblotting.

Filipin staining and Nile-Red staining

HL-7702 cells were seeded in 24-well plates at the density of 5×10^4 cells/well and maintained in 5% CO_2 at 37 °C for 24 h. Then cells were transfected with siRNA for 48 h. After that, cells were incubated in medium D for 24 h, or medium D with the indicated concentrations of corylin for 16 h. After the treatment, 50 μg/ml fillipin or 0.5 μg/ml Nile-Red were used to stain the treated cells for 30 min at 24 °C. The filipin- and Nile-Red stained cells were analyzed with EVOS FL Auto microscope

(life technologies).

Cholesterol and TG measurement

For measurement of intracellular TC and TG, the cells were cultured in six-well plates and collected in 1 ml PBS. 100 μl of the total cell suspension were transferred to a new tube and centrifuged at 1,000 g for 5 min at 4 °C. Then this portion of the cells were lysed in lysis buffer (RIPA lysis buffer) and used for protein quantification. The remaining cell suspension was used for lipid extraction. After centrifugation at 1000 g for 5 min at 4 °C, the collected cells were mixed with 1ml of chloroform/methanol (2:1, v/v) adequately on a shaker for 3 h at 24 °C. Then 500 μl NaCl (0.1 M) was added into each reaction tube and mixed thoroughly, followed by centrifugation at 3700 rpm for 10 min. the lower organic phase was transferred and evaporated to dryness. The residual liquid was re-suspended in 50 μl of 1% Triton-X 100 in absolute ethanol, and the concentrations of TC or TG were measured using the TC or TG determination kit according to the manufacturer's instructions, respectively (Shanghai, China). For measurement of liver TC and TG, 40–50 mg of liver tissue was homogenized in 0.5 ml PBS. About 5 μl of the total homogenates were used for protein quantification. About 0.4 ml homogenates were mixed with 1.6 ml of chloroform/methanol (2:1, v/v) adequately for lipid extraction. The following experimental procedures were identical with measurement of hepatic TC and TG.

Virtual screening

The crystal structures of HSP90β (3PRY) and HSP90α (3Q6M) were downloaded from the RCSB protein data bank for molecular docking. The crystallographic water

in 3PRY and 3Q6M was removed. The compounds were sketched by Maestro and processed by LigPrep under its default parameters. Molecular docking was performed using Glide6.9 (Schrödinger, LLC, New York, NY, USA) in XP mode. Default values for other parameters were adapted. We screened the top 12 compounds with the largest difference in the binding energy between 3PRY and 3Q6M (Table S2). The difference of binding energy of the compound with 3PRY and 3Q6M is at least greater than 1.

Akt kinase activity measurement

HL-7702 cells were treated with corylin or 17-AAG. After that, the cells were lysis and obtained the protein buffer. The Akt kinase activity was detected by AKT ELISA Kit (ZciBio). The experimental procedure is carried out according to the Kit instructions.

In silico molecular docking research

To analyze the binding affinities of corylin to HSP90β and the possible binding sites, an in silico protein-ligand docking software AutoDock 4.2 program was applied. The docking steps were performed as the follows: Crystal structure file of HSP90β (3PRY) was downloaded from the RCSB protein data bank; (2) Deletion of unnecessary substructures and water molecules; (3) Hydrogen atoms were added to HSP90β; (4) Gasteiger charges were calculated for each atom of HSP90β; (5) Run Autogrid to get grid maps; (6) Run 100 times to generate docked conformations by Lamarckian genetic algorithm (LGA) and obtain former 20 conformations with the lowest binding energies for statistical analysis. The interaction figures of ligands to HSP90β were

generated and the results of docking were recorded with binding orientation ratio and binding energy. The percentage binding orientation ratio of a ligand to HSP90β was calculated as % binding orientation ratio = EC/TC × 100, where EC is the number of effective conformations of ligands binding to the function domain of XOD, and TC is the total number of statistical conformations.

Recombinant protein production

Recombinant human EGFP-HSP90α, EGFP-HSP90β, EGFP-HSP90β(1-218), EGFP-HSP90β(1-276), EGFP-HSP90β(1-602), EGFP-HSP90β(276-602), EGFP-HSP90β (W312A/N375A/N436A) were expressed in Escherichia coli BL21 (DE3) strain as C-terminal His-6-tagged fusion proteins by using the pET28a expression system (Novagen). The C-terminal tagged (His) 6 fusion proteins were purified by Ni^{2+}-agarose affinity chromatography.

ATPase activity of HSP90β measurement

We used purified HSP90α and HSP90β (wild type or triple mutation) proteins to detect their chaperone activity *in vitro* by QuantiChrom™ ATPase Assay Kit (BioAssay Systems). The experimental procedure is carried out according to the Kit instructions.

Microscale thermophoresis analysis

Corylin, 17-AAG, AP-III-a4, compound A or neobavaisoflavone were titrated in different concentrations to purified recombinant human EGFP-HSP90β, EGFP-HSP90α, His-ENO1 EGFP-HSP90β(1-218), EGFP-HSP90β(1-276), EGFP-HSP90β(1-602), EGFP-HSP90β (276-602) or

EGFP-HSP90β(W312A/N375A/N436A) proteins. The reaction was performed in 50 mM Hepes, 50 mM NaCl, 0.01% Tween-20 and 2 mM $MgCl_2$. Then the samples were incubated in room temperature for 5 min before analyzing by microscale thermophoresis. A NanoTemper Monolith Instrument (NT.115) was used for measuring thermophoresis. In this instrument, an infra blue-Laser (IB-Laser) beam couples into the path of light (i.e. fluorescence excitation and emission) with a dichroic mirror and is focused into the sample fluid through the same optical element used for fluorescence imaging. The IB laser is absorbed by the aqueous solution in the capillary and locally heats the sample with a $1/e^2$ diameter of 25 μm. Up to 24 mW of laser power where used to heat the sample, without damaging the biomolecules. To analyze the thermophoresis of a sample, ten microliters were transferred in a glass capillary (NanoTemper, hydrophilic treated). Thermophoresis of the protein in presence of varying concentrations of compound was analyzed for 30 seconds. Measurements were performed at room temperature and standard deviation was calculated from three independent experiments.

Photo-affinity labeling and pull down experiment

Photo-affinity-linker-coated (PALC) agarose beads and corylin-immobilized beads were prepared according to previous reported method. The protein extracts of HepG2 (2mg in 1ml NETN lysis buffer, 0.1% NP-40, 0.5 mM EDTA, 20 mM Tris-HCl, 150 mM $NaCl_2$, protease inhibitor cocktail, pH=8.0) were first pre-incubated in the presence (3 replications) or absence (3 replications) of corylin (finally 0.5 μM) at 4 °C overnight, and then 12 μl of prewashed corylin-immobilized beads were added to each

sample, and incubated at 4 °C for an additional 4 h. Subsequently, the beads were washed with lysis buffer for 3 times and eluted with 1×SDS-loading buffer (20 μl), then boiled for 5 min at 95 °C. The eluted proteins were analyzed according to digestion in gel method, and further analyzed by an EASY-nLC 1000 nano-flow LC instrument coupled to a Q Exactive quadrupole-orbitrap mass spectrometer (Thermo Fisher Scientific).

Pull down experiment

Briefly, corylin beads and blank beads (corylin free control) were prepared according to previous reported method. Then 12 μl of prewashed corylin-immobilized beads (2 group, 3 replications) and blank beads (3 replications) were incubated with HepG2 cell protein extracts (2 mg in 1ml NETN lysis buffer, 0.1% NP-40, 0.5 mM EDTA, 20 mM Tris-HCl, 150 mM NaCl, protease inhibitor cocktail, pH=8.0) at 4 °C for 4 h. Subsequently, the beads were washed with lysis buffer for 3 times, and one group of corylin beads and blank beads were eluted with 100 μM corylin (in 40 μl PBS) and the other group of corylin beads were eluted with 40 μl PBS at 37 °C for 10 min. The eluted proteins were analyzed according to digestion in gel method, and further analyzed by an EASY-nLC 1000 nanoflow LC instrument coupled to a Q Exactive quadrupole-orbitrap mass spectrometer (Thermo Fisher Scientific). The screening principle is that statistical analyses were done with student's *t*-test vs blank ($p^{*}<0.05$), the ratio of corylin-1/blank is greater than 1.2, and the ratio of coyrlin-1/corylin-2 is greater than 4.5. (blank: Intensity of the proteins, eluted by 100 μM corylin (PBS buffer) from blank beads; corylin_1: Intensity of the proteins, eluted by 100 μM

corylin (PBS buffer) from corylin linked beads; corylin_2: Intensity of the proteins, eluted by PBS buffer from corylin linked beads).

Metabolic measurements

After receiving different treatments for 6 weeks, mice from each group were acclimated in a comprehensive lab animal monitoring system (Columbus Instruments, Columbus, OH) for 24 h according to the instructions of the manufacturer. After mice adapted to the metabolic chamber, volume of O_2 consumption and CO_2 production were continuously recorded over a 24 h period. RQ equals volumes of CO_2 released/volumes of O_2 consumed.

Adenovirus-mediated RNAi in mice liver

The adenoviral expression kit from Life Technologies was utilized to construct the adenovirus-producing plasmids containing a gene of shRNA HSP90β or LacZ. The adenovirus vectors were digested with Pac I, then transfected the 293A producer cell line in a 6-well-plate. The media was placed with DMEM containing 10% FBS and 1% penicillin/streptomycin the next day. The cells transferred to 10 cm tissue culture dishes after the transfection for 24 h. We replaced the culture media with fresh media every 2–3 days until cytopathic effect (CPE) was observed. We collected the cells when 80% CPE were observed and harvested adenovirus by repeatedly freezing at –80 °C and thawing at 37 °C for 4 times. We centrifuged cell lysates at 2,000g for 30 min at 25 °C and stored the supernatant containing adenovirus particles at –80 °C. The adenoviruses were packaged in HEK293 cells and purified with CsCl ultracentrifugation. The viruses were tittered and administrated via caudal vein

injection (5×10^9 pfu viruses per mouse).

HSP90β shRNA sequence:

5’-CACCGCACTGCGAGACAACTCTACACGAATGTAGAGTTGTCTCGCAGTG C-3’

5’-AAAAGCACTGCGAGACAACTCTACATTCGTGTAGAGTTGTCTCGCAGTG C-3’

LacZ shRNA sequence:

5’-CACCGCTACACAAATCAGCGATTTCGAAAAATCGCTGATTTGTGTAG-3’;

5’-AAAACTACACAAATCAGCGATTTTTCGAAATCGCTGATTTGTGTAGC-3’

Fecal cholesterol and TG measurements

After receiving different treatments for 6 weeks, mice were kept into the metabolic chambers for 24 h to collect the feces which were lyophilized and ground up. About 250 mg mashed feces were extracted with 4 ml of methanol: chloroform (1:2, v/v) twice. The supernatants were pooled. Exactly 100 μl was removed and evaporated to dryness. TG was measured with determination kit. Then other 4 ml mixture of 5 N KOH: ethanol (10: 90, v/v) were added and heated at 70 °C for 1 h. After cooling to room temperature, 2 ml ultrapure water was added and saturated with sodium chloride, followed by solvent extraction with 3 ml hexane twice. The extracts were dried and re-dissolved with 50 μl hexane contained 50 μl 5-a-cholestane (1 mg/ml) was added. Then GC-MS analyses were performed with an Agilent 7890B gas chromatograph interfaced to an Agilent 5977A mass-selective detector and equipped with HP-5ms Ultra Inert (30 m×250 mm×0.25 μm) column (Agilent Technologies, USA). The

temperatures of the injector, interface, and ion source were 280 °C, 280 °C, and 230 °C, respectively. Helium was used as the carrier gas at a flow rate of 0.7 ml/min in constant flow mode.

The weight of fat analysis by NMR

To determine the fat content of animals, after six-week treatment, the mice were scanned with the minispec TD-NMR designed for experimental animals (Bruker, Germany), the fat content was calculated according to the measurement between the solid and liquid parts of the sample.

Serum and liver lipid determination

Serum TC and TG levels were measured according to the manufacturer's instructions (Kehua, China), and LDL-C and HDL-C levels were determined by the kits (Njjcbio, China). Liver tissues were homogenized and centrifuged. Supernatants were collected, and TC and TG were determined.

Glucose tolerance and insulin tolerance tests

Glucose tolerance tests (GTT) and insulin tolerance tests (ITT) were performed on mice fasted overnight with free access to water. Mice were injected with 0.75 U/kg insulin (Sigma) by i. p. or gavaged with 2 g/kg glucose (Sigma) by i. g. Glucose levels were measured from tail blood at 15, 30, 60, or 120 min after the injection. All animals were sacrificed 3 days after glucose tolerance or insulin tolerance tests, and blood and liver were harvested. Area under the curve (AUC) was calculated to quantify the GTT and ITT results.

Histological analysis of liver, adipose and aortas

Liver, WAT, BAT and aortas were fixed in 4% paraformaldehyde at 4 °C overnight and embedded in paraffin wax. Paraffin sections (5 µm) were cut and mounted on glass slides for H&E staining. Cryosections of livers were stained by oil red O and counterstained with hematoxylin to visualize the lipid droplets.

Immunohistochemistry

Immunohistochemistry was carried out using 3 µm thick sections fixed in 4% paraformaldehyde. After deparaffinization, rehydration and antigen retrieval, sections were incubated in blocking buffer containing 10% normal goat serum in PBS. Sections were incubated with described antibodies followed by washing and incubated with HRP-tagged goat anti-rabbit secondary antibody. Samples were subsequently rinsed in wash buffer and incubated in diaminobenzidine (Sigma, St. Louis, MO) for 5 minutes and counterstained in hematoxylin. Tissue slides were scored in a blinded fashion. No staining was observed with negative control rabbit anti-IgG antibody. The images were measured blindly by one observer using Image-Pro Plus (Media Cybernetics, Silver Spring, USA).

Analysis of atherosclerotic lesions

To quantify atherosclerosis along the entire aorta, the aortic tree was dissected out and the lesions were stained with oil red O for 6 min, destained with 80% ethanol for 3 min. Sudanophilic lesions were assessed by computer-assisted image analysis.

Supplementary Figure 1

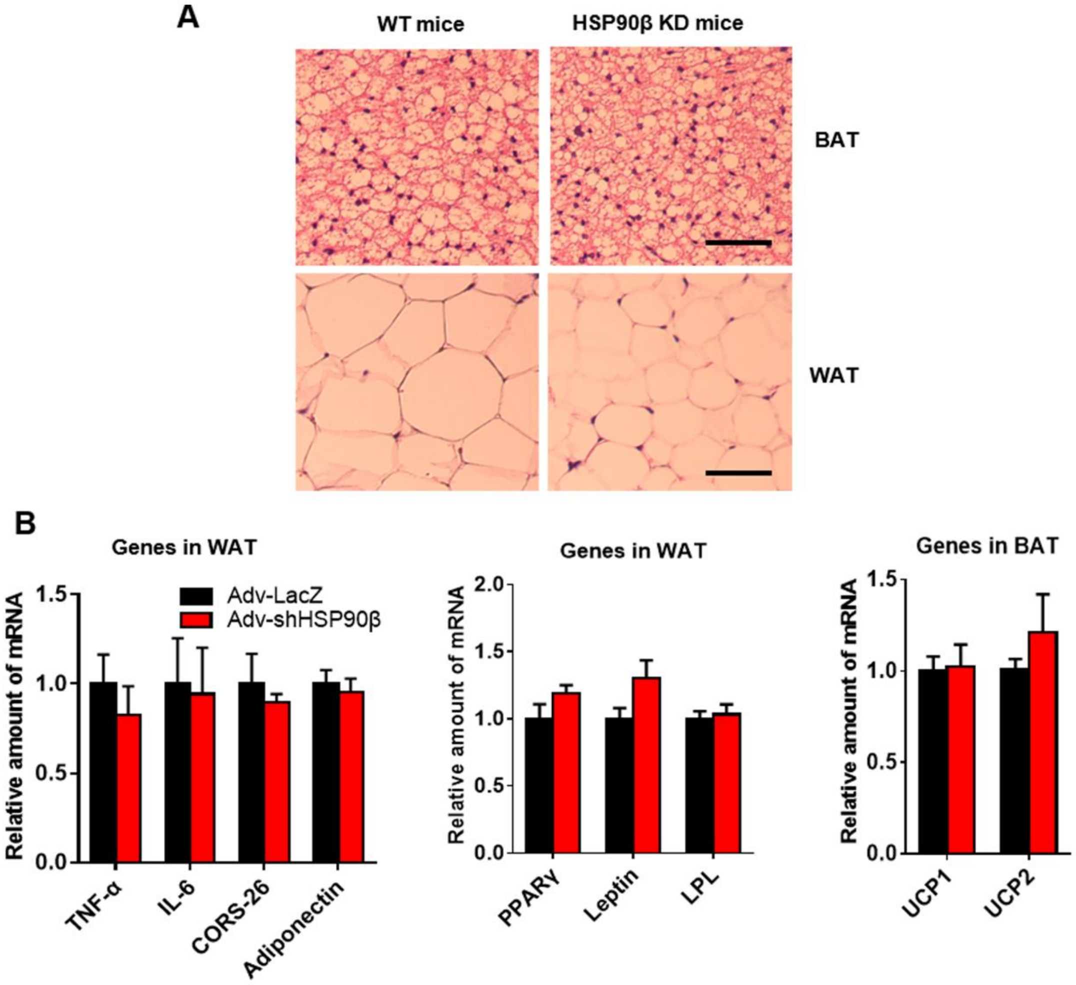

Supplementary Figure 1. Pathological staining and gene expression in adipocytes of Adv-shHSP90β treated mice

Male C57BL/6J mice (6 weeks) were randomly grouped (10 mice each group). Mice were allowed *ad libitum* access to water and high fat diet (HFD). After four weeks, mice were intravenously injected with titer of 5×10^9 adenovirus expressing the shRNA targeting HSP90β or the shRNA targeting LacZ. HFD was still administrated to mice for additional 14 days. Then, mice were sacrificed and subjected to various analysis. (A) H&E staining of BAT or WAT sections. (B) The total RNAs from mice adipocytes were prepared and subjected to qRT-PCR. Mouse GAPDH was used as the

internal control. Error bars are represented as mean ± SEM. Statistical analysis was done with student's *t*-test. *$p < 0.05$, **$p < 0.01$, ***$p < 0.001$ *vs* control adenovirus shRNA.

Supplementary Figure 2

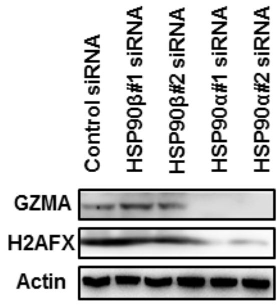

Supplementary Figure 2. The effect of corylin, HSP90β or HSP90α knockdown on their client proteins

HL-7702 hepatocytes were transfected with siRNA targeting HSP90β or HSP90α, then incubated in medium B for 48 h, whole cell extracts underwent immunoblotting with indicated antibodies.

Supplementary Figure 3

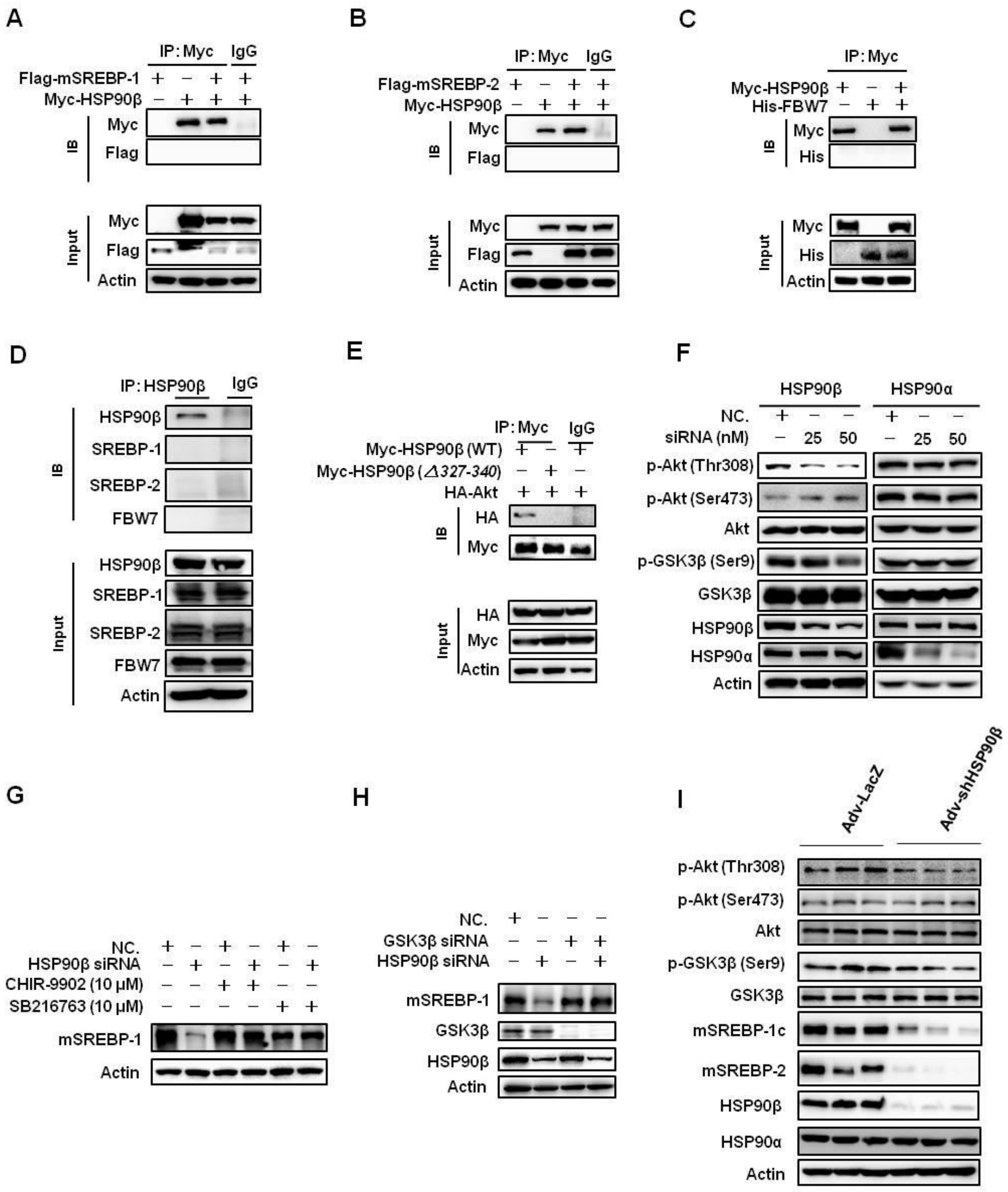

Supplementary Figure 3. HSP90β promotes the mSREBP ubiquitin proteasome degradation dependent on Akt-GSK3β signaling pathway

(A-C) 293T cells were transfected with or without the indicated plasmids for 24 h. Immunobloting for indicated proteins after immunoprecipitation of myc from 293T cells. (D) HL-7702 cells were transfected with or without the indicated plasmids for 24 h. Immunobloting for indicated proteins after immunoprecipitation of HSP90β from HL-7702 cells. (E) 293T cells were transfected with or without the indicated plasmids for 24 h. Immunobloting for indicated proteins after immunoprecipitation of myc from 293T cells. (F) HL-7702 cells were treated with indicated HSP90 siRNA for 48 h, cells were lysed and the expression of proteins was measured by WB. (G) HL-7702 cells were treated with HSP90β siRNA for 48 h. After that, the cells were switched to medium D treated with GSK3β inhibitor CHIR-9902 (10 μM) or SB216763 (10 μM) for 1 h, the cells were switched to medium D supplemented with CHIR-9902 (10 μM) or SB216763 (10 μM) for 4 h. (H) HL-7702 cells were transfected with indicated siRNA for 48 h, after the treatment, the whole cell extracts underwent immunoblotting with indicated antibodies. (I) Male C57BL/6J mice (6 weeks) were randomly grouped (10 mice each group). Mice were allowed *ad libitum* access to water and high fat diet (HFD). After four weeks, mice were intravenously injected with titer of 5×10^9 adenovirus expressing the shRNA targeting HSP90β or the shRNA targeting LacZ. HFD was still administrated to mice for additional 14 days. Then, mice were sacrificed and the total protein from mice liver were prepared, and subjected to immunoblot.

Supplementary Figure 4

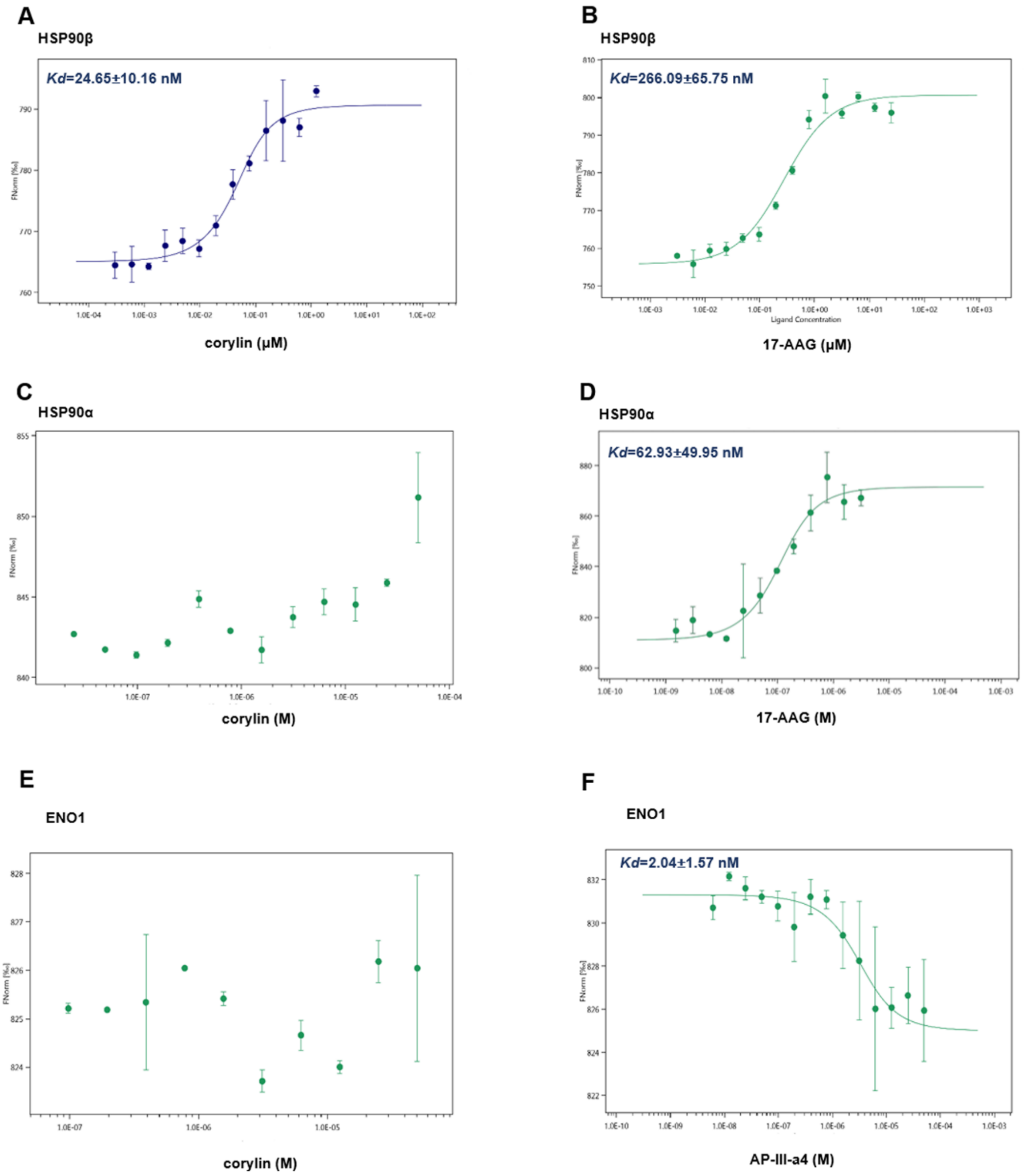

Supplementary Figure 4. The interaction between corylin and recombination protein HSP90β, HSP90α and ENO1.

(A and B) The EGFP-HSP90β concentration was kept constant at 20 nM and the small molecule was titrated from 10 μM corylin (A) or 100 μM 17-AAG (B). The interaction between mutant proteins and 17-AAG or corylin was detected by microscale thermophoresis (MST). (C and D) The EGFP-HSP90α concentration was kept constant at 20 nM and the small molecule was titrated from 50 μM corylin (C) or 50 μM 17-AAG (D). (E and F) ENO1 concentration was kept constant at 20 nM and the small molecule was titrated from 50 μM corylin (E) or 50 μM AP-III-a4 (F).

Supplementary Figure 5

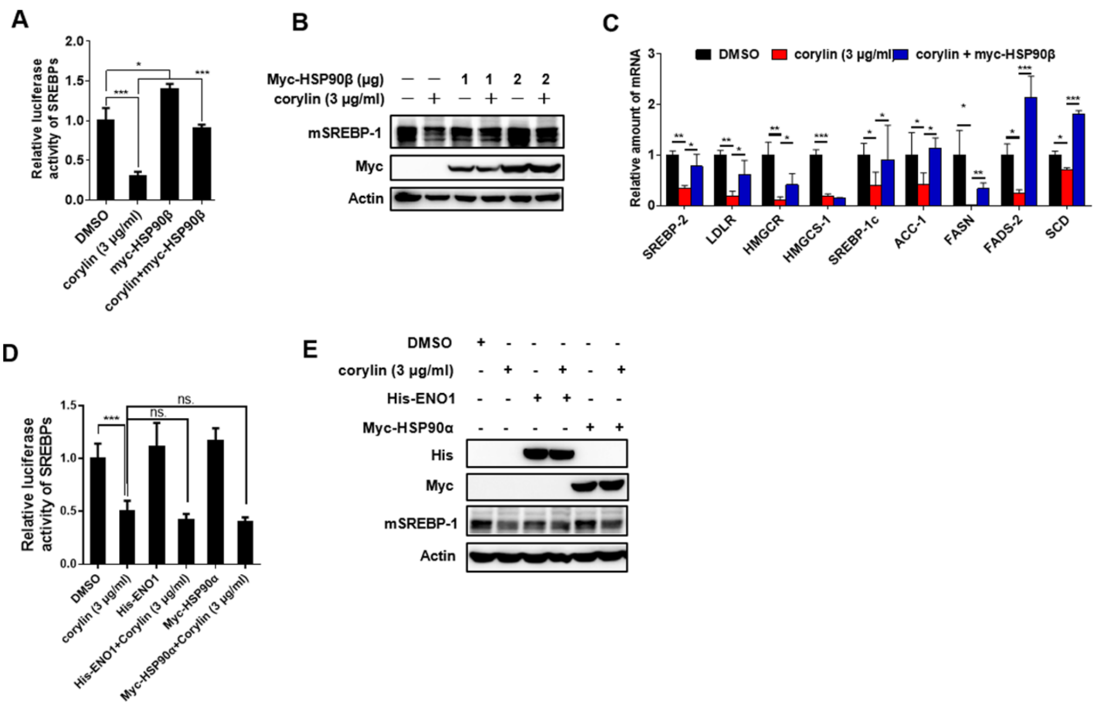

Supplementary Figure 5. Reverse of corylin effect by overexpressing HSP90β.

(A) HL-7702/SRE-Luc cells were transfected with myc-HSP90β for 24 h. The cells were depleted of sterols by incubating in medium D for 24 h, and then switched to medium D containing corylin or not for 4 h. The cells were lysed and luciferase activity was measured. (B and C) HL-7702 hepatocytes were transfected with indicated concentration of myc-HSP90β plasmid for 24 h. The cells were incubated in medium D for 24 h, and switched to medium D containing corylin for 4 h, (B) the whole cell extracts underwent immunoblotting with indicated antibodies. (C) The expression of various genes was analyzed by qRT-PCR. (D and E) HL-7702/SRE-Luc cells were transfected with His-ENO1 or Myc-HSP90α overexpression plasmids for 24 h. The cells were depleted of sterols by incubating in medium D for 24 h, and then

switched to medium D containing corylin or not for 4 h. (D) The cells were lysed and luciferase activity was measured. (E) The whole cell extracts underwent immunoblotting with indicated antibodies. Error bars are represented as mean ± SEM. Statistical analyses were done with one-way ANOVA (Dunnett's post test) (A, C and D). *$p < 0.05$, **$p < 0.01$, ***$p < 0.001$ vs DMSO or corylin.

Supplementary Figure 6

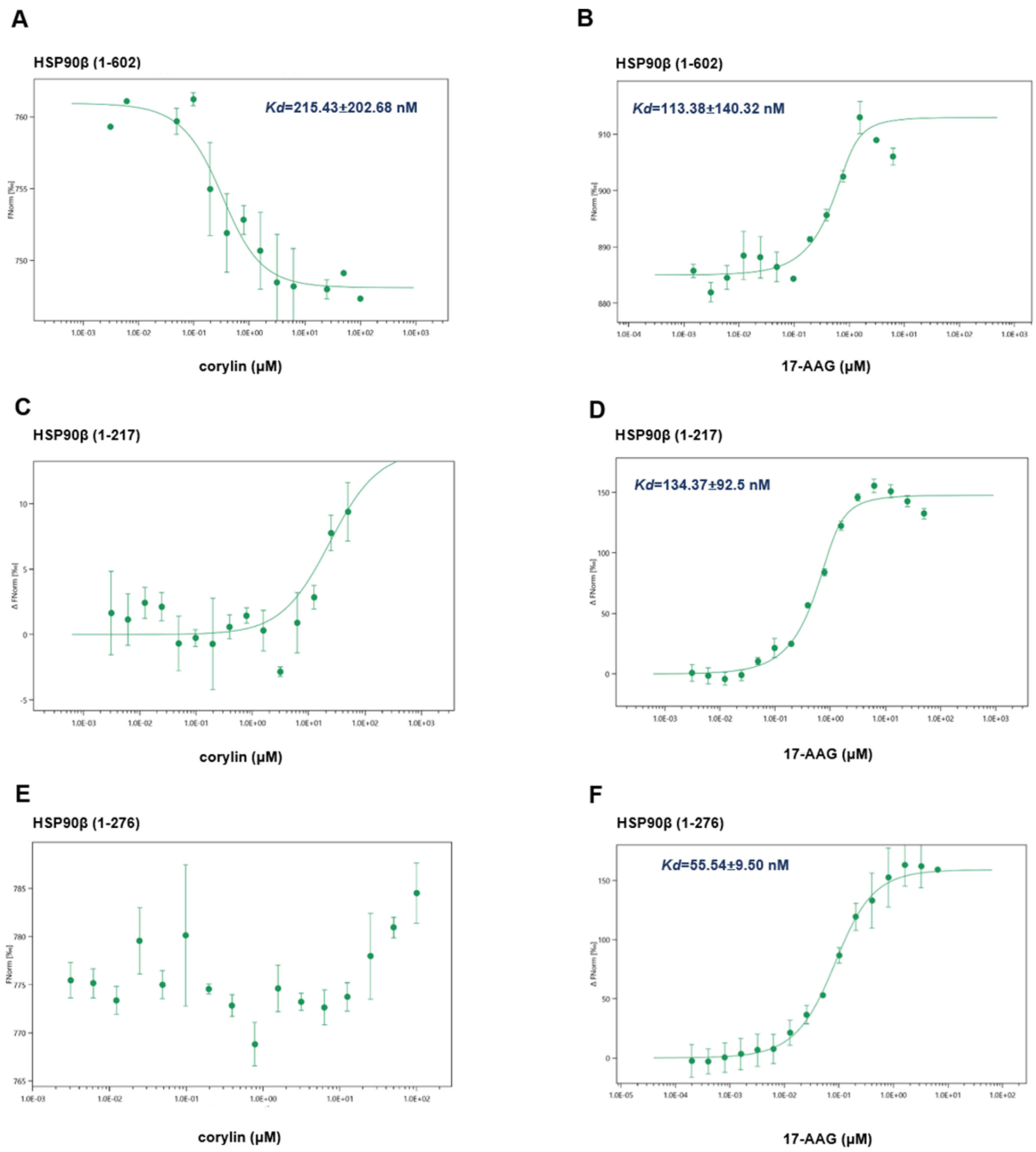

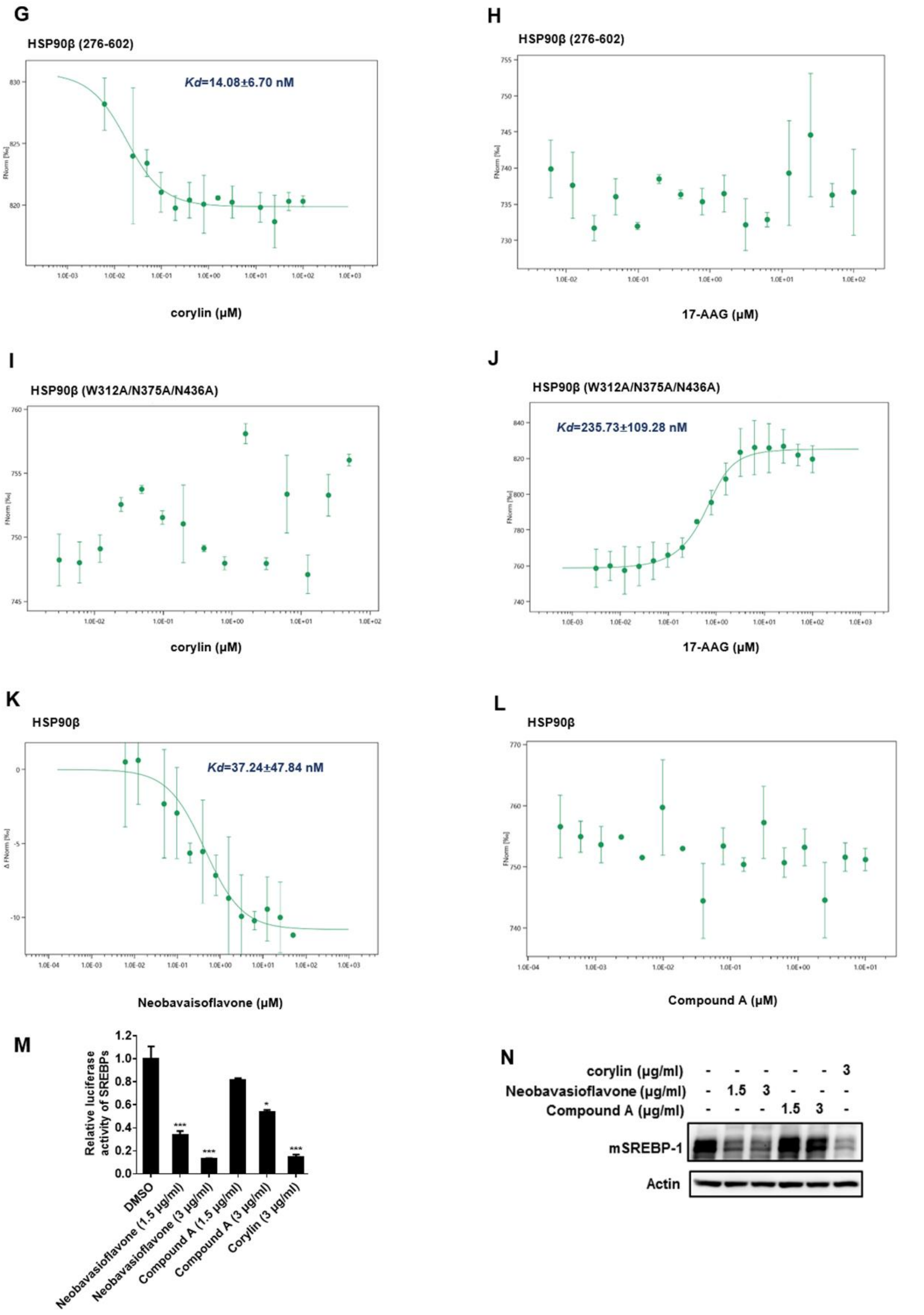

Supplementary Figure 6. The interaction between recombination protein HSP90β or the mutant protein of HSP90β and corylin.

(A and B) The interaction between EGFP-HSP90β (1-602) and corylin (A) or 17-AAG (B). (C and D) The interaction between EGFP-HSP90β (1-217) and corylin (C) or 17-AAG (D). (E and F) The interaction between EGFP-HSP90β (1-276) and corylin (E) or 17-AAG (F). (G and H) The interaction between EGFP-HSP90β (276-602) and corylin (G) or 17-AAG (H). (I and J) The interaction between EGFP-HSP90β (W312A/N375A/N436A) and corylin (I) or 17-AAG (J). (K and L) The interaction between EGFP-HSP90β and neobavaisoflavone (K) or Compound A (L) was detected by microscale thermophoresis (MST). The interaction between proteins and compounds was detected by microscale thermophoresis. (M) The HL-7702/SRE-Luc cells were depleted of sterols by incubating in medium D for 24 h, and then switched to medium D containing corylin, compound A, or neobavaisoflavone for 4 h. The cells were lysed and luciferase activity was measured. (N) The whole cell extracts underwent immunoblotting with indicated antibodies. Error bars are represented as mean ± SEM. Statistical analyses were done with one-way ANOVA (Dunnett's post test). *$p < 0.05$, **$p < 0.01$, ***$p < 0.001$ vs DMSO.

Supplementary Figure 7

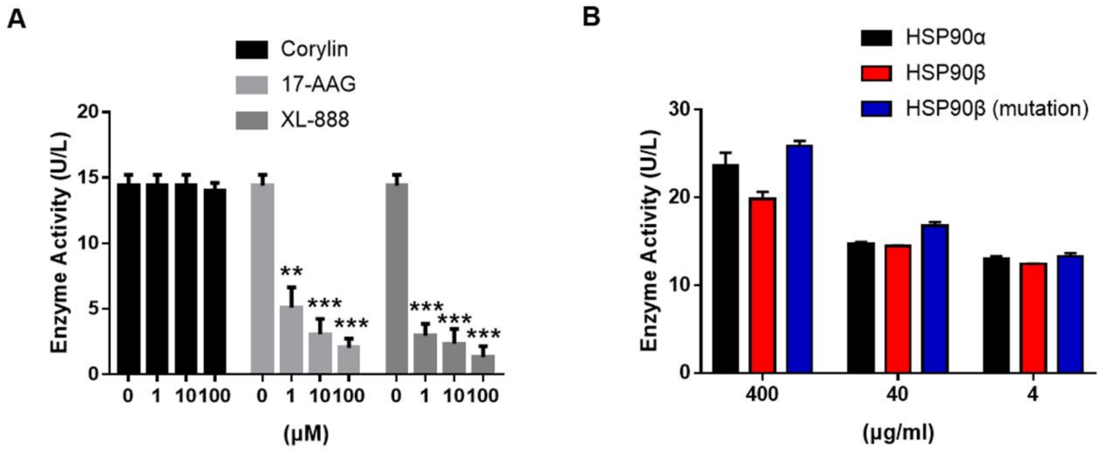

Supplementary Figure 7. Triple mutation or corylin did not affect HSP90β ATPase activity

(A) The HSP90β concentration was kept at 4 μg/ml and then incubated corylin, 17-AAG or XL-888 with different concentration for 30 min. after that, the reaction was stopped and detected the ATPase activity. (B) The HSP90α, HSP90β and HSP90β (W312A/N375A/N436A) proteins were purified and diluted to different concentrations (400, 40 and 4 μg/ml). Those proteins were used to detect the ATPase activity. Error bars are represented as mean ± SEM. Statistical analysis was done with one-way ANOVA (Dunnett's post test). *p < 0.05, **p < 0.01, ***p < 0.001 *vs* control.

Supplementary Figure 8

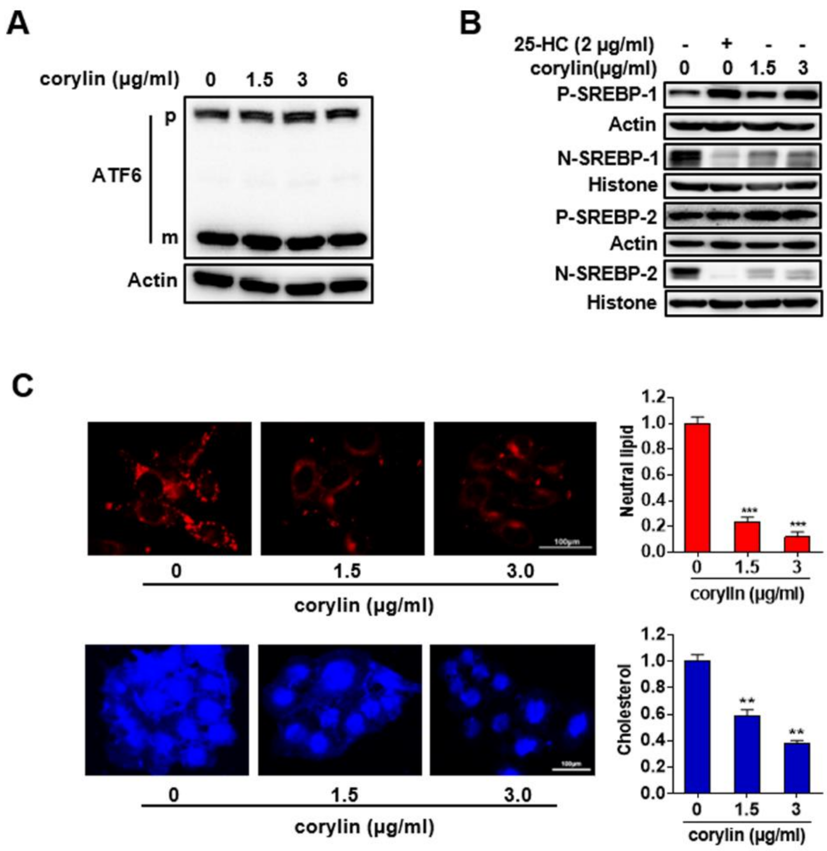

Supplementary Figure 8. Corylin reduced mSREBPs levels and lipid content in HL-7702 cells.

(A) The siRNA targeting HSP90α treated HL-7702 hepatocytes incubated in medium B for 72 h, and the cell viability was measured by MTT assay. (C and D) HL7702 cells were depleted of sterols by incubating in medium D for 24 h, and then switched to medium D containing indicated concentration of corylin for 16 h. (B) The whole cell extracts underwent immunoblotting with ATF6, (C) the nuclear separation extracts underwent immunoblotting with indicated antibodies. (D) The treated cells were fixed and stained with Nile-Red or filipin. Quantification of the cellular neutral lipids or cholesterol was analyzed by Image-Pro Plus. Error bars are represented as mean ± SEM. Statistical analyses were done with one-way ANOVA (Dunnett's post

test). *$p < 0.05$, **$p < 0.01$, ***$p < 0.001$ vs DMSO.

Supplementary Figure 9

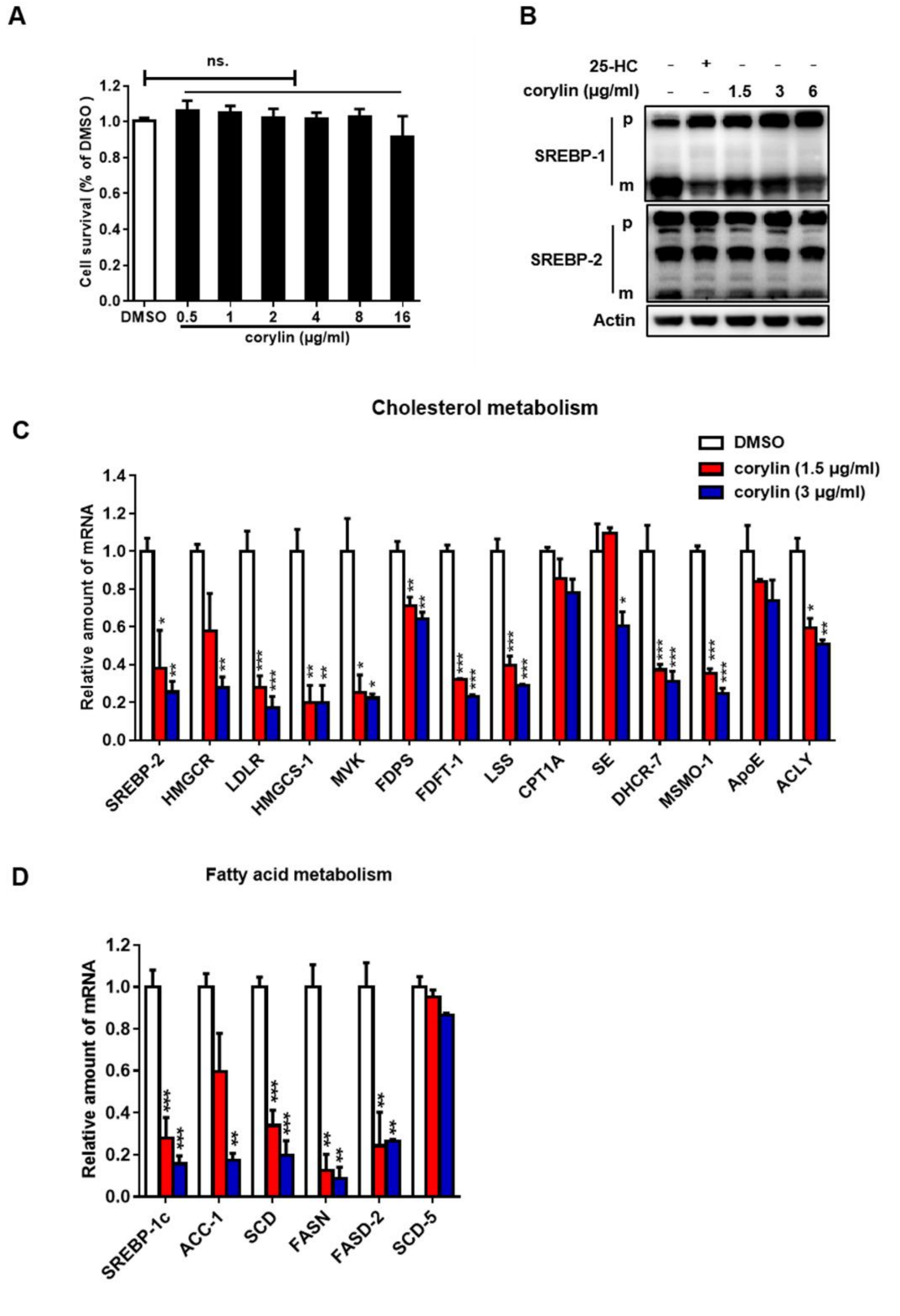

Supplementary Figure 9. Corylin suppressed SREBPs activity in primary human hepatocytes.

(A) HH cells were treated with increasing concentrations of corylin for 24 h, and cell viability was measured by MTT assay. (B) HH cells were switched to medium D containing 25-HC, or increasing concentrations of corylin. After incubation for 4 h, whole cell proteins were underwent IB with indicated antibodies. (C and D) HH cells were treated with 1.5 or 3 μg/ml corylin for 4 h. The cells were then cracked in Trizol reagents, and RNAs were extracted. The expression of various genes was analyzed by reverse transcription followed by qRT-PCR. All experiments were repeated three times. Statistical analyses were done with one-way ANOVA (Dunnett's post test). $^{*}p < 0.05$, $^{**}p < 0.01$, $^{***}p < 0.001$ *vs* DMSO.

Supplementary Figure10

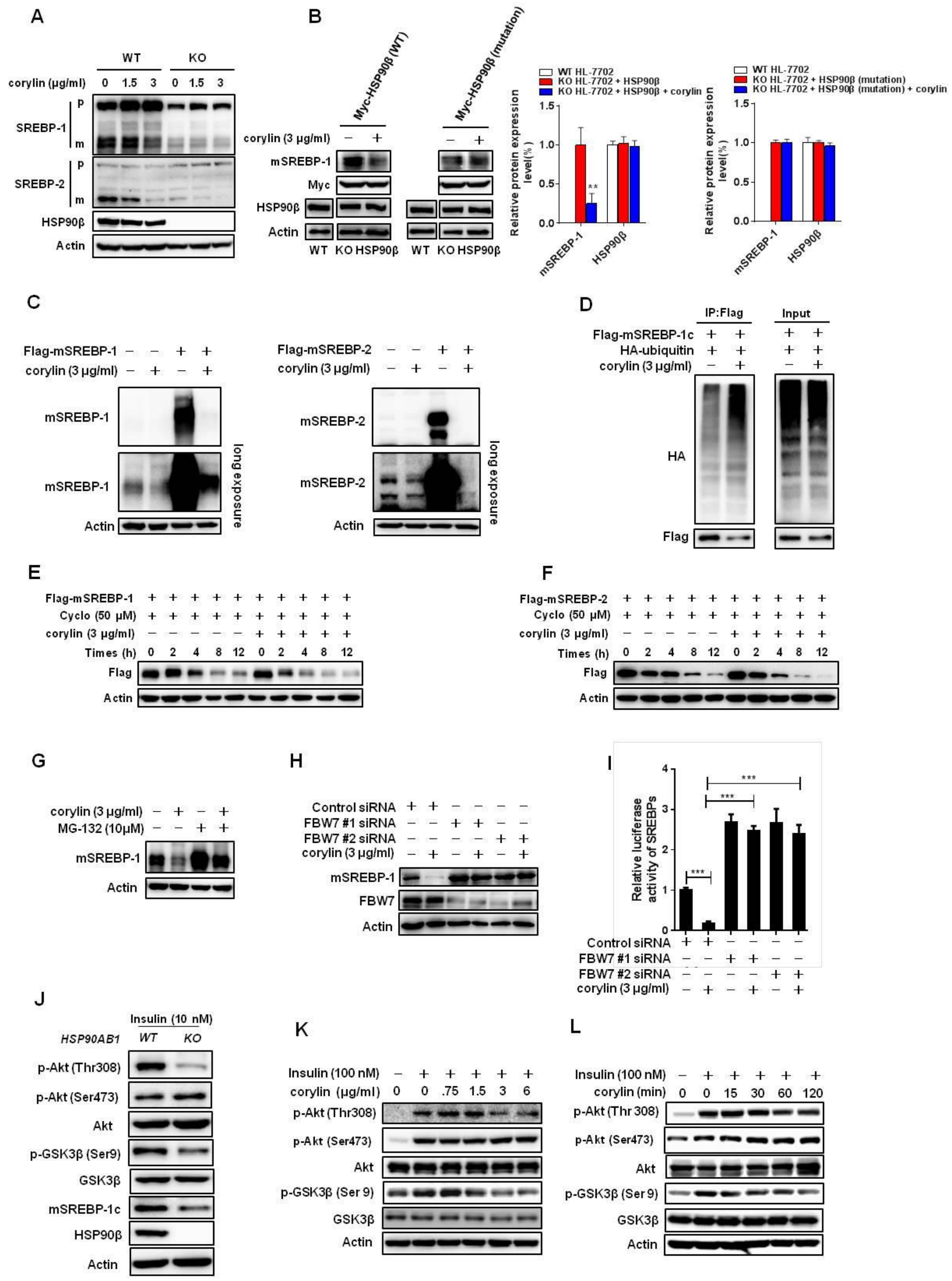

Supplementary Figure 10. Corylin promotes mSREBPs ubiquitin proteasome degradation through Akt-GSK3β pathway

(A) The HL-7702/WT cells and HL-7702/KO cells were incubated in medium D for 24 h, and switched to medium D containing indicated concentration of corylin for 4 h, the whole cell extracts underwent immunoblotting with indicated antibodies. (B) HL-7702/KO cells were transfected with Myc-HSP90β (wild type, WT) or Myc-HSP90β (mutation, W312A/N375A/N436A) for 48 h. The level was comparable to endogenous HSP90β expression level. After that, the cells were treated with corylin for another 4 h, whole cell extracts underwent immunoblotting with indicated antibodies. (C) HL-7702 cells were transfected with flag-mSREBP-1 or flag-mSREBP-2 plasmids and cultured for 24 h. The cells were switched to medium D supplemented with vehicle, or 3 μg/ml corylin for 4 h. (D) 293T cells were transfected with flag-mSREBP-1 and HA-ubiquitin for 24 h, after the treatment, the cells were incubated with medium D containing corylin for another 4 h. Cells were lysed and pulled down by flag antibody. (E and F) HL-7702 cells were transfected with flag-mSREBP-1 (E) or flag-mSREBP-2 (F) plasmids and cultured for 24 h. After incubation with 50 μM cycloheximide for 1 h, the cells were switched to medium D supplemented with 50 μM cycloheximide plus vehicle, or 3 μg/ml corylin for incubation as indicated periods of time. (G) HL-7702 cells were switched to medium D treated with 10 μM MG-132 for 1 h, the cells were switched to medium D supplemented with 10 μM MG-132 plus vehicle, or 3 μg/ml corylin for 4 h. (H) HL-7702 cells were transfected with siRNA FBW7#1 or siFBW7 #2 for 48 h, after

the treatment, the cells were switched to medium D treated with 3 μg/ml corylin for 4 h. (I) HL-7702/SRE-Luc cells were transfected with treated with siRNA FBW7#1 or siFBW7 #2 for 48 h, luciferase activity was measured (K and L) HL-7702 cells were incubated with insulin for 1 h. After the treatment, the cells were treated with indicated concentrations of corylin for 2 h, or with corylin (3 μg/ml) for indicated periods of times. After that, the whole cell extracts underwent immunoblotting with indicated antibodies. (J) HSP90β KO cells were treated with 10 nM insulin 2 h, whole cell extracts underwent immunoblotting with indicated antibodies. Error bars are represented as mean ± SEM. Statistical analyses were done with one-way ANOVA (Dunnett's post test). $^{**}p < 0.01$， $^{***}p < 0.001$ *vs* control.

Supplementary Figure 11

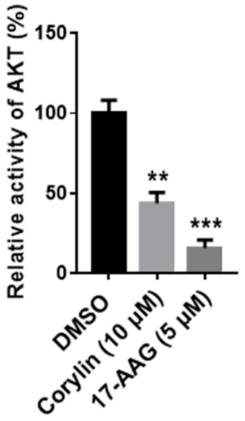

Supplementary Figure 11. The effect of corylin or 17-AAG on Akt kinase activity

HL7702 cells were treated with 10 μM corylin or 5 μM 17-AAG for 4 h, and then the Akt kinase activity was measured accordingly. Error bars are represented as mean ± SEM. Statistical analysis was done with one-way ANOVA (Dunnett’s post test). **p < 0.01, ***p < 0.001 *vs* DMSO.

Supplementary Figure 12

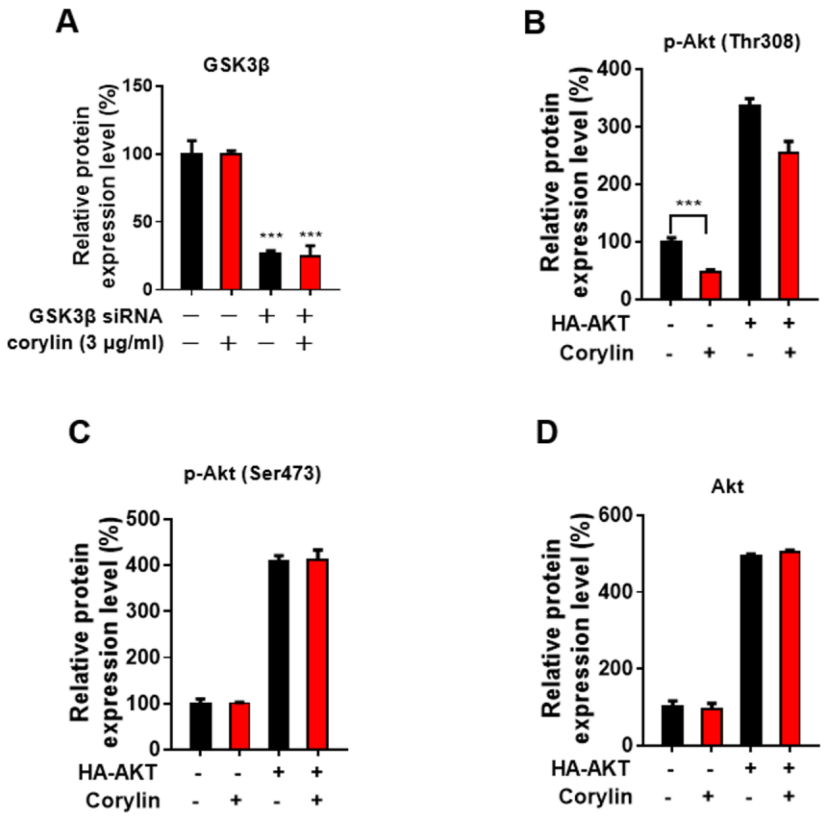

Supplementary Figure 12. GSK3β knockdown and AKT overexpression effect.

(A) siRNA effect on GSK3β was analyzed by qRT-PCR. (B) Relative T308, (C) S473 phosphorylation and total Akt level in Akt overexpression cells.

Supplementary Figure 13

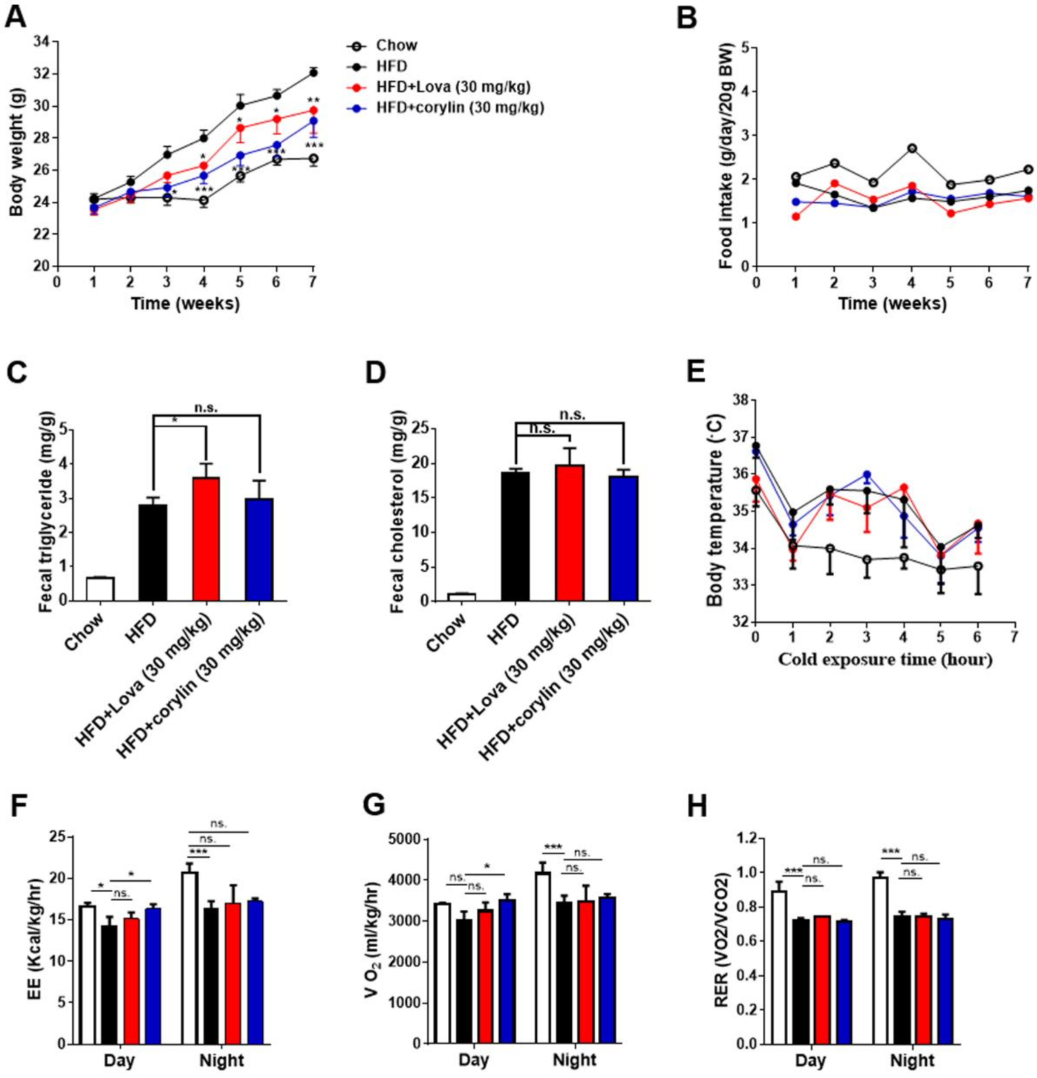

Supplementary Figure 13. Effects of corylin on energy metabolism in HFD-induced mice

Male C57BL/6J mice at 6 weeks of age were randomly grouped (n=6). Mice were allowed *ad libitum* access to water and different types of diets. Vehicle (0.5% CMC-Na), corylin (30 mg/kg), or lovastatin (30 mg/kg) was administrated to mice by gastric irrigation every day. After 6 weeks treatment, the mice were placed into metabolic chambers to measure oxygen consumption, CO_2 production, etc. Feces were collected to measure fecal cholesterol and triglyceride. (A) Body weight. (B) Food intake during the 6 week experiments. (C and D) Effects of corylin or lovastatin on the fecal TG (C) and TC (D) levels. (E) Body temperature of different groups of mice at different time points after cold exposure (4 °C). (F) Energy expenditure was measured as kilocalories per kilogram lean mass per hour. (G) Oxygen consumption was measured as milliliters volume oxygen per kilogram lean mass per hour. (H) Substrate utilization. This is expressed by respiratory exchange ratio (RER), which is the volume ratio of oxygen consumed versus CO_2 exhaled. Error bars are represented as mean ± SEM. Statistical analyses were done with two-way ANOVA (Bonferroni's test) (A, B, and C) or one-way ANOVA (Dunnett's post test) (D-H). *p < 0.05, **p < 0.01, ***p < 0.001 *vs* HFD.

Supplementary Figure 14

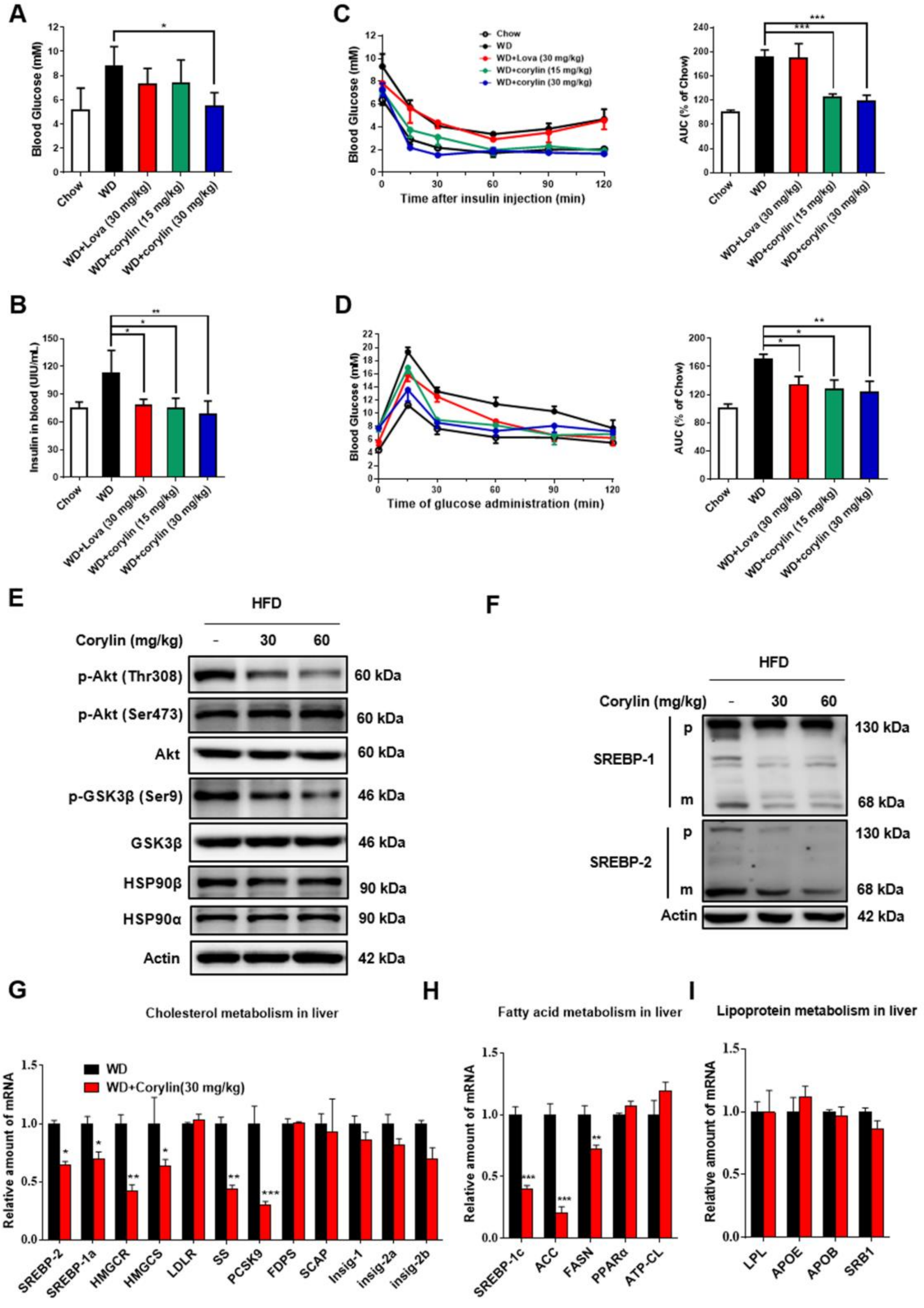

Supplementary Figure 14. Corylin improves insulin resistance and suppressed SREBP activity in WD-fed mice

(A and B) Blood glucose (A) and blood insulin (B) in WD-fed mice were improved by corylin treatment. (C) Effect of corylin on glucose tolerance in WD fed mice was determined by glucose tolerance test (GTT). Quantification of the area under the curve (AUC) from the GTT. (D) Effect of corylin on insulin resistance in WD-fed mice determined by insulin tolerance test (ITT). Quantification of the AUC of the ITT. (E-F) After 6 weeks treatment, equal amounts of total proteins from livers of 3 mice in each group were subjected in immunoblotting with indicated antibodies. (G-I) For each group, equal amounts of total RNA from tissues of 3 mice were analyzed. Gene expression in liver (G-I) were detected by qRT-PCR. Error bars are represented as mean±SEM. Statistical analyses were done with one-way ANOVA (Dunnett's post test). *p < 0.05, **p < 0.01, ***p < 0.001 vs WD.

Supplementary Figure 15

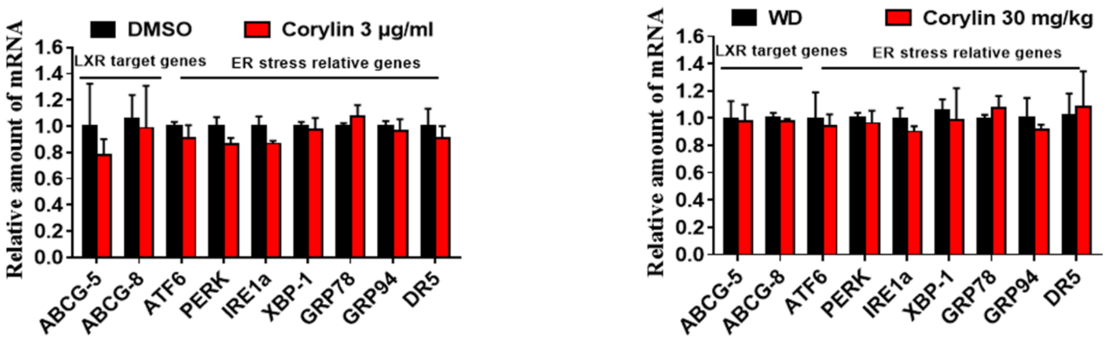

Supplementary Figure 15. Corylin neither activated the LXR pathway nor induced ER stress

(A) HL-7702 cells were depleted of sterols by incubating in medium D for 24 h, and then switched to medium D containing 3 μg/ml corylin for 4 h. The cells were then cracked in Trizol reagents, and RNAs were extracted. (B) WD-fed mice were treated with vehicle or corylin, mice liver were collected and the expression of various genes was analyzed by qRT-PCR. Error bars are represented as mean ± SEM. Statistical analyses were done with student’s *t*-test. **p < 0.01， ***p < 0.001 *vs* DMSO.

Supplementary Figure 16

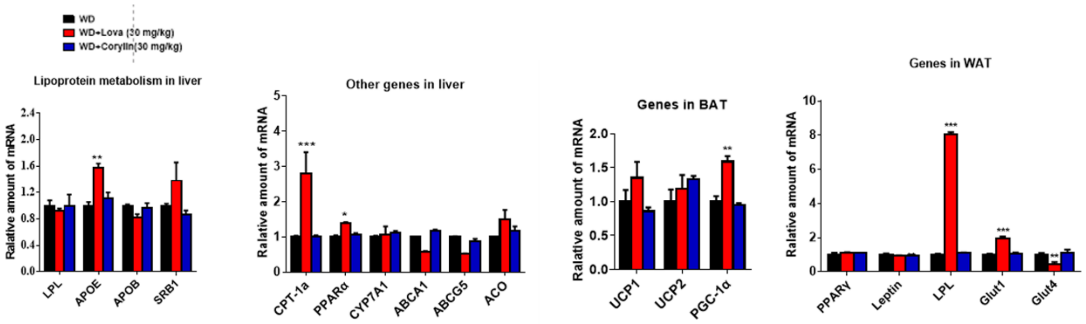

Supplementary Figure 16. Corylin does not affect lipid metabolic genes expression in BAT and WAT

Male C57BL/6J mice at 6 weeks of age were randomly grouped (n=6). Mice were allowed ad libitum access to water and different types of diets (WD, western-type diet). Vehicle (0.5% CMC-Na), corylin (15 or 30 mg/kg), or lovastatin (30 mg/kg) was administrated to mice by gastric irrigation every day. After 6 weeks treatment, the mice were sacrificed, liver, BAT, WAT tissues were collected and mRNAs were analyzed by qRT-PCR. The expression of various genes was analyzed by qRT-PCR. Error bars are represented as mean ± SEM. Statistical analyses were done with student's *t*-test. **p < 0.01， ***p < 0.001 *vs* DMSO.

Supplementary Figure 17

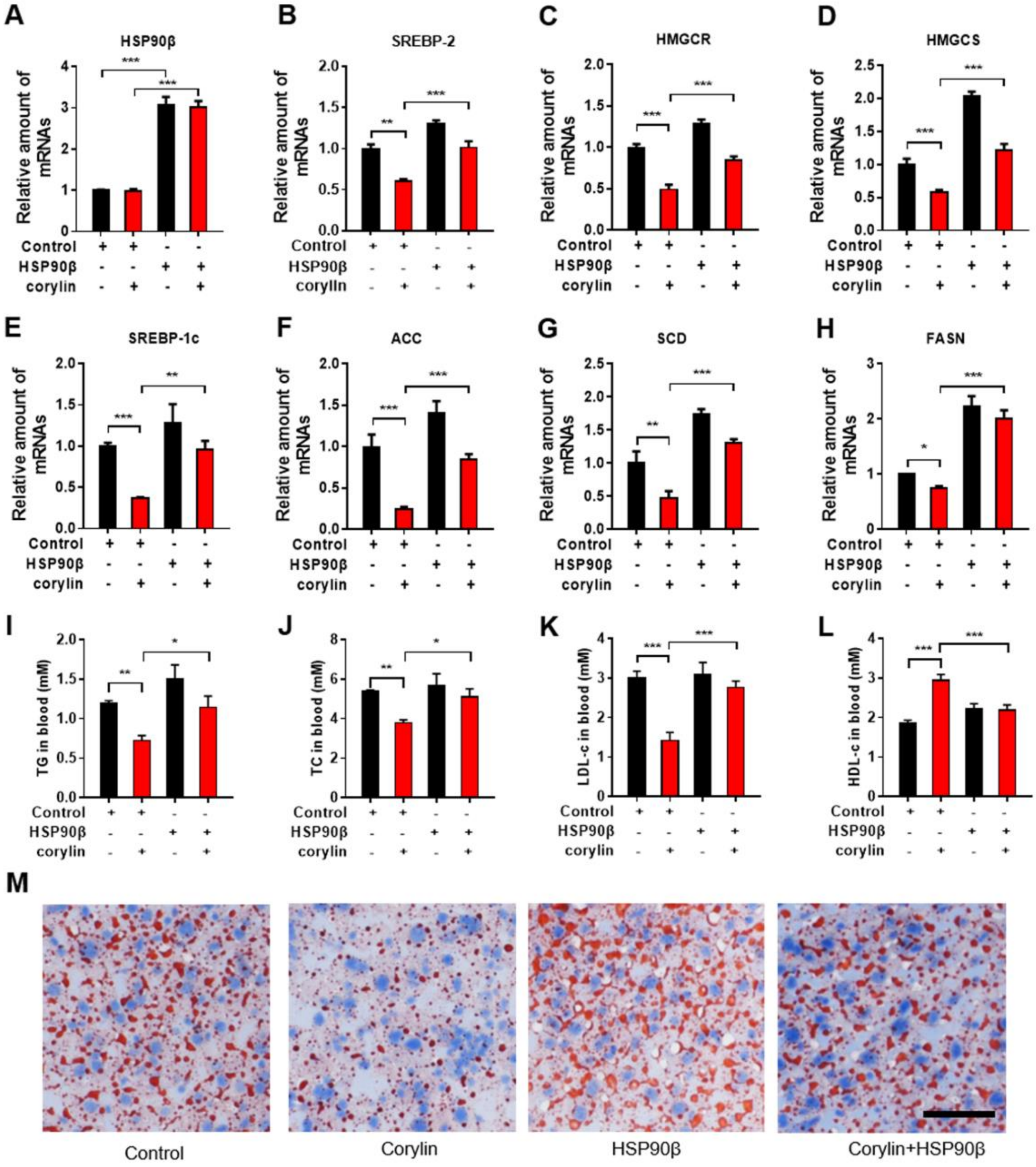

Supplementary Figure 17. The *in vivo* Effects of corylin are dependent on HSP90β

Male C57BL/6J mice at 6 weeks of age were randomly grouped (6 per group). Mice were allowed *ad libitum* access to water and High-fat diet (HFD). Vehicle, 30 mg/kg/day of corylin was administrated to mice by gastric irrigation once daily. After four-week treatment, mice were intravenously injected with titer of 5×10^{8} adenoviral

virus expressing the plasmid for HSP90β or control plasmid. Vehicle or 30 mg/kg/day of corylin was still administrated to mice by gastric irrigation once daily for 14 days. Then, mice were sacrificed and subjected to various analyses. (A-H) SREBPs and their target gene expression in mice liver. (I) Blood total cholesterol (TC) levels, (J) triglyceride levels (TG), (K) low density lipoprotein cholesterol (LDL-c) levels, and (L) high density lipoprotein cholesterol (HDL-c) levels were analyzed after HSP90β overexpression. (M) the oil red staining of liver. Error bars represent standard deviations. Statistical analysis was done with one-way ANOVA (Dunnett's post test). $*p < 0.05$, $**p < 0.01$, $***p < 0.001$ *vs* control.

Supplementary Figure 18

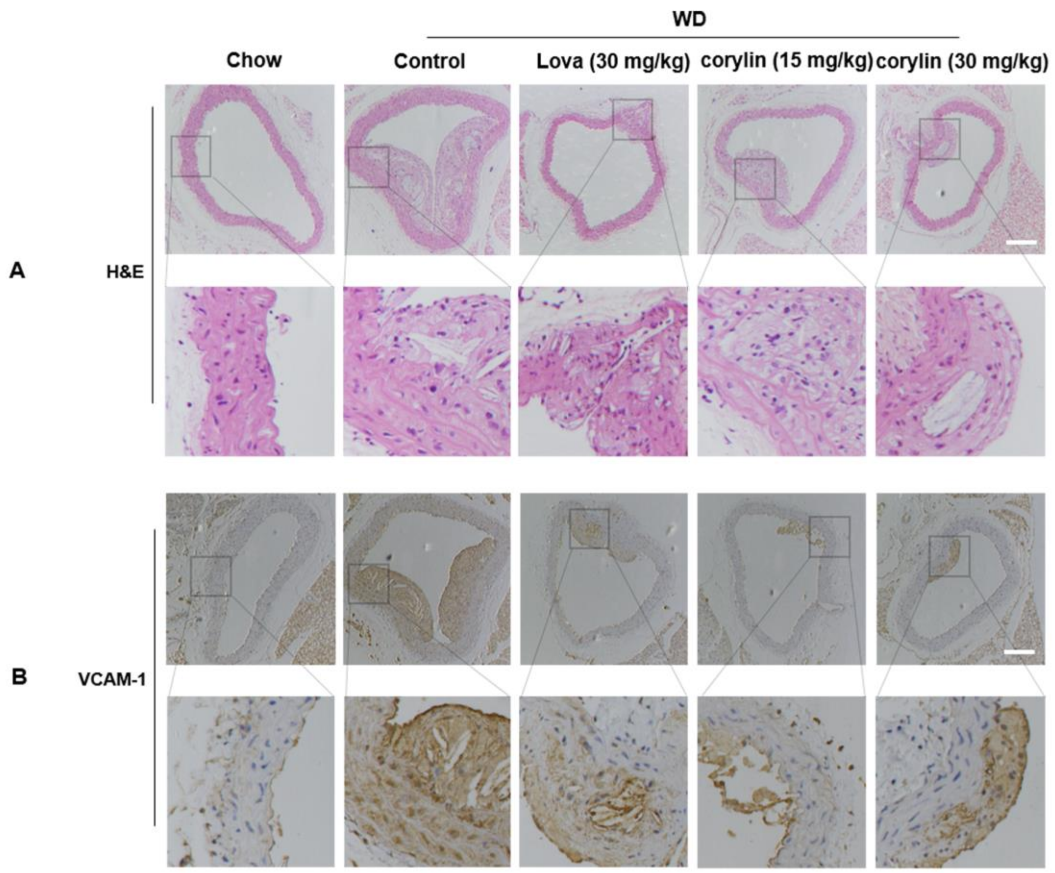

Supplementary Figure 18. Corylin decreases the atherosclerosis development in ApoE$^{-/-}$ mice (continue to Fig. 7)

Six-week-old male ApoE$^{-/-}$ mice were randomly grouped (twelve each group) and fed with WD supplemented with vehicle, corylin (30mg/kg) or lovastatin (30 mg/kg) for 16 weeks. After the treatment, the mice were sacrificed. The aortic tree was isolated and subjected to various analyses as described below. (A) Histological analysis of aortas. (B) Aortas were immunohistochemically stained with VCAM-1 antibody.

Supplementary Table 1. Characteristics of NAFLD patients and NAFLD-free donors

Characteristics of NAFLD patients

Subject	Age/gender	BMI (kg/m^2)	Histological grading			Glucose (mmol/L)	Insulin (pmol/ml)	TG (mmol/L)	TC (mmol/L)
			Steatosis	Inflammation	Fibrosis				
1	30/F	49.98	3	1	2	4.8	43.91	4.02	6.36
2	50/F	37.07	2	1	2	6.37	18.4	2.14	3.57
3	28/M	36.59	2	0	2	6.31	23.79	1.23	5.31
4	69/F	44.40	2	1	1	-	9.08	2.51	5.56
5	33/F	38.07	2	0	1	5.94	20.16	1.9	4.65
6	31/M	39.86	2	1	1	5.48	45.32	2.59	4.76
7	53/F	31.25	2	0	2	4.72	10.73	1.38	4.89
8	54/F	33.75	2	1	2	5.99	43.21	1.14	3.31
9	32/M	30.19	2	1	0	6.06	16.60	0.93	4.61
10	51/F	38.81	2	1	0	6.96	19.19	2.26	5.05
11	47/M	25.61	1	0	2	6.99	25.75	1.23	2.55
12	46/F	32.00	3	1	1	5.54	39.63	3.76	4.56
13	57/M	32.74	1	1	0	8.14	10.21	1.74	4.28
14	54/M	33.14	2	0	1	5.91	46.12	1.14	6.23
15	16/M	37.60	3	1	1	3.83	-	1.57	3.97
16	37/F	26.70	2	1	0	5.17	-	2.02	4.55
17	45/M	29.14	2	1	1	5.39	42.14	1.20	5.31
18	48/F	36.43	2	2	2	4.85	43.17	1.39	4.21
19	35/M	39.26	3	1	0	3.57	-	1.95	4.26
20	41/F	35.19	2	1	1	5.19	-	2.42	5.31

Characteristics of NAFLD-free donors

Subject	Age/gender	BMI (kg/m^2)	Histological grading			Glucose (mmol/L)	Insulin (pmol/ml)	TG (mmol/L)	TC (mmol/L)
			Steatosis	Inflammation	Fibrosis				
1	40/M	-	0	0	0	4.72	-	0.81	2.34
2	59/M	-	0	0	0	5.06	-	0.59	3.38
3	38/M	-	0	0	0	6.11	-	0.69	3.11
4	69/F	-	0	0	0	4.77	-	0.77	2.51
5	43/F	-	0	0	0	4.24	-	0.38	2.38

Supplementary Table 2. The results of virtual screening

Compound	The binding energy (kcal/mol)		The difference between	Inhibition rate of SRE-Luc
	3pry (HSP90AB1)	3q6m (HSP90AA1)	3pry and 3q6m	(%)
**corylin**	**-9.42**	**-4.823**	**4.597**	**90.6**
Swertisin	-7.851	-4.759	3.092	57.0
Rhynchophylline	-5.174	-2.24	2.934	-2
Syringin	-7.241	-4.67	2.571	23.0
agnuside	-7.417	-4.986	2.431	-9
Prunetin	-6.057	-3.85	2.207	56.2
Vicenin-2	-8.347	-6.4	1.947	27.1
Oleuropein	-8.02	-6.277	1.743	-1
Levodopa	-8.242	-6.582	1.66	44.3
Cynarin	-9.595	-7.948	1.647	47.2
Alkannin	-7.089	-5.713	1.376	29.1
Linarin	-9.245	-7.965	1.28	21.2

Supplementary Table 3. The proteins pulled down by corylin in HepG2 cells

See the Excel Table (Supplementary Table 3) for details.

Supplementary Table 3. The proteins pulled down by corylin in HepG2 cells

Protein names	Gene names	blank_1	blank_2	blank_3	corylin_1_1	corylin_1_2	corylin_1_3	corylin_2_1	corylin_2_2	corylin_2_3	P value	ation of corylin-1/blank-	ation of corylin-1/corylin-2
Actin, cytoplasmic 1;Actin, cytopla	ACTB;ACTG1;ACTG2;A	1.153E+09	1.19E+09	1.07E+09	1.28E+09	1.12E+09	1.46E+09	8.94E+08	2.47E+08	2.51E+08	0.2359	1.132014072	2.772767094
Heat shock cognate 71 kDa protein	HSPA8	458220000	4.59E+08	3.26E+08	3.4E+08	4.11E+08	5.13E+08	1.51E+08	13228000	17244000	0.9255	1.017561792	6.984178719
Heat shock protein HSP 90-beta	**HSP90AB1**	**65205000**	**66852000**	**59262000**	**1.18E+08**	**1.12E+08**	**81912000**	**33717000**	**0**	**0**	**0.0245**	**1.633669421**	**9.269863867**
Stress-70 protein, mitochondrial	HSPA9	155230000	1.54E+08	1.64E+08	95538000	1.06E+08	1.55E+08	30413000	0	0	0.1166	0.75354146	11.72222405
Annexin A2;Putative annexin A2-lil	ANXA2;ANXA2P2	53958000	61736000	47172000	92783000	70796000	43898000	62047000	19228000	27014000	0.3736	1.273912296	1.915956376
L-lactate dehydrogenase B chain	LDHB	117600000	1.11E+08	1.02E+08	92525000	93744000	1.48E+08	97065000	48378000	44525000	0.9435	1.012486737	1.758132949
Tubulin beta chain;Tubulin beta-3 ch	TUBB;TUBB3	81192000	78633000	93123000	91075000	1.01E+08	1.32E+08	59582000	0	0	0.1458	1.28210146	5.44300292
Peroxiredoxin-1	PRDX1	157930000	1.66E+08	1.92E+08	64089000	78313000	75051000	0	0	0	0.0007	0.421829292	∞
78 kDa glucose-regulated protein	HSPA5	58116000	59446000	54215000	52983000	71766000	1.01E+08	0	0	0	0.2662	1.315245929	∞
**Heat shock protein HSP 90-alph**	**HSP90AA1**	**32701000**	**31268000**	**39367000**	**51046000**	**58099000**	**47222000**	**0**	**0**	**0**	**0.0121**	**1.513189982**	**∞**
Putative elongation factor 1-alpha-l	EEF1A1P5;EEF1A1;EEI	51891000	48686000	56035000	47736000	54450000	37097000	0	0	14673000	0.3412	0.889350752	9.492469161
**Alpha-enolase**	**ENO1**	**30376000**	**35872000**	**45304000**	**46761000**	**56509000**	**51163000**	**33793000**	**0**	**0**	**0.0415**	**1.384403686**	**4.569970112**
Elongation factor 1-gamma	EEF1G	38023000	43957000	45510000	42699000	41927000	58620000	0	0	0	0.4249	1.123586164	∞
Eukaryotic initiation factor 4A-I;Eu	EIF4A1;EIF4A2	42932000	45765000	39153000	42649000	36533000	45447000	28936000	0	0	0.7442	0.974806414	4.307056953
Heat shock 70 kDa protein 1B;Heat	HSPA1B;HSPA1A	59542000	59041000	63968000	41912000	48995000	50527000	21329000	0	0	0.0113	0.774764312	6.631065685
Glyceraldehyde-3-phosphate dehydr	GAPDH	55155000	55083000	50433000	38530000	50991000	43328000	0	0	0	0.0791	0.826838695	∞
Ubiquitin-60S ribosomal protein L4(	UBA52;RPS27A;UBB;UI	30042000	33194000	40349000	32460000	42821000	15432000	18667000	28358000	38570000	0.6471	0.875734904	1.059793212
Tubulin alpha-1C chain;Tubulin alph	TUBA1C;TUBA1A;TUE	30317000	34707000	30101000	28969000	32595000	34698000	0	0	0	0.9091	1.011952694	∞
Guanine nucleotide-binding protein s	GNB2L1	22540000	26585000	32509000	25273000	30813000	33879000	32727000	0	0	0.5285	1.102053066	2.748953464
Desmoplakin	DSP	182890000	2.16E+08	1.61E+08	23728000	19278000	52049000	14809000	0	0	0.0015	0.169883652	6.418731852
60 kDa heat shock protein, mitocho	HSPD1	18805000	22331000	17390000	18546000	23679000	34479000	0	0	0	0.2756	1.310597	∞
Serpin B12	SERPINB12	22876000	22334000	27678000	14798000	12138000	15317000	0	0	0	0.0075	0.579697618	∞
Junction plakoglobin	JUP	102350000	83672000	81520000	13655000	9664600	44159000	35699000	0	7824600	0.0062	0.252216848	1.550391052
Histone H4	HIST1H4A	40551000	36828000	27327000	12940000	27952000	69350000	0	0	1.32E+08	0.9228	1.052871851	0.837832497
Leukocyte elastase inhibitor	SERPINB1	11334000	10781000	14471000	12310000	9927200	14963000	0	0	0	0.946	1.016787842	∞

Description of Data:

blank: Intensity of the proteins, eluted by 100 μM corylin (PBS buffer) from blank beads. Experiment was carried out in three duplicates, namely, blank_1, blank_2, blank_3.

corylin_1: Intensity of the proteins, eluted by 100 μM corylin (PBS buffer) from corylin linked beads. Experiment was carried out in three duplicates, namely, corylin_1_1, corylin_1_2, corylin_1_3.

corylin_2:Intensity of the proteins,eluted by PBS buffer from corylin linked beads.Experiment was carried out in three duplicates,namely,corylin_2_1,corylin_2_2,corylin_2_3.

P value: Student t-test was used to analyze significance between Corylin_1 and blank.

Supplementary Table 4. Nucleotide sequences of gene-specific primers used for qRT-PCR, related to the experimental procedures

Specifies	Gene name	Sequence of forward and reverse primers(5' to 3')
Mus musculus	GAPDH	TGTGTCCGTCGTGGATCTGA<br>CCTGCTTCACCACCTTCTTGAT
	SREBP-1a	GGCCGAGATGTGCGAACT<br>TTGTTGATGAGCTGGAGCATGT
	SREBP-1c	GGAGCCATGGATTGCACATT<br>GGCCCGGGAAGTCACTGT
	SREBP-2	GCGTTCTGGAGACCATGGA<br>ACAAAGTTGCTCTGAAAACAAATCA
	SCAP	ATTTGCTCACCGTGGAGATGTT<br>GAAGTCATCCAGGCCACTACTAATG
	HMGCS	GCCGTGAACTGGGTCGAA<br>GCATATATAGCAATGTCTCCTGCAA
	HMGCR	CTTGTGGAATGCCTTGTGATTG<br>AGCCGAAGCAGCACATGAT
	FDPS	ATGGAGATGGGCGAGTTCTTC<br>CCGACCTTTCCCGTCACA
	SS	CCAACTCAATGGGTCTGTTCCT<br>TGGCTTAGCAAAGTCTTCCAACT
	LDLR	AGGCTGTGGGCTCCATAGG<br>TGCGGTCCAGGGTCATCT
	ACC	TGACAGACTGATCGCAGAGAAAG<br>TGGAGAGCCCCACACACA
	FASN	GCTGCGGAAACTTCAGGAAAT<br>AGAGACGTGTCACTCCTGGACTT
	SCD5	ATTCCTCTTCTCTGTCCCTC<br>TCCACCACCACAGCACTC
	ATP-CL	CCGGAGACCCCTTAGATCGA<br>TAGCCTGTAAAAGATTTCTGCAAACC
	ApoB	CGTGGGCTCCAGCATTCTA<br>TCACCAGTCATTTCTGCCTTTG
	ApoE	GCTGGGTGCAGACGCTTT<br>TGCCGTCAGTTCTTGTGTGACT

Mus musculus	ABCA1	CGTTTCCGGGAAGTGTCCTA<br>GCTAGAGATGACAAGGAGGATGGA
	ABCG5	TGGATCCAACACCTCTATGCTAAA<br>GGCAGGTTTTCTCGATGAACTG
	Insig-1	TCACAGTGACTGAGCTTCAGCA<br>TCATCTTCATCACACCCAGGAC
	Insig-2a	CCCTCAATGAATGTACTGAAGGATT<br>TGTGAAGTGAAGCAGACCAATGT
	Insig-2b	CCGGGCAGAGCTCAGGAT<br>GAAGCAGACCAATGTTTCAATGG
	SR-B1	TGGACAAATGGAACGGACTC<br>GTGAAGCGATACGTGGGAAT
	LPL	CTTCTTGATTTACACGGAGGT<br>ATGGCATTTCACAAACACTG
	PPARα	TCTGTGGGCTCACTGTTCT<br>AGGGCTCATCCTGTCTTTG
	HSP90AB1	CCTGGGAACCATTGCTAAGTCT<br>GCCCGATCATGGAGATGTCT
	HSP90AA1	CGGACGCTCTGGATAAAATCC<br>TCCTTCCCCGAGTCCAGTTT
	PPAR-γ	GGCTGAGGAGAAGTCACACTCTG<br>AAATCTTGTCTGTCACACAGTCCTG

Homo sapiens	GAPDH	GGAGCGAGATCCCTCCAAAAT<br>GGCTGTTGTCATACTTCTCATGG
	SREBP-2	AACGGTCATTCACCCAGGTC<br>GGCTGAAGAATAGGAGTTGCC
	HMGCR	TGATTGACCTTTCCAGAGCAAG<br>CTAAAATTGCCATTCCACGAGC
	LDLR	ACCAACGAATGCTTGGACAAC<br>ACAGGCACTCGTAGCCGAT
	HMGCS-1	CTCTTGGGATGGACGGTATGC<br>GCTCCAACTCCACCTGTAGG
	HMGCS-2	GGCGGGTCCTGCAAGTGAAGA<br>GGGGAGCAGGAGGGATTGTAGAAA
	MVK	GGAGCAAGGTGATGTCACAAC<br>CGGCAGATGGACAGGTATAAGT
	FDPS	TGTGACCGGCAAAATTGGC<br>GCCCGTTGCAGACACTGAA

Homo sapiens	FDFT-1	CCACCCCGAAGAGTTCTACAA<br>TGCGACTGGTCTGATTGAGATA
	LSS	GTACGAGCCCGGAACATTCTT<br>CGGCGTAGCAGTAGCTCAT
	Insig-1	CCTGGCATCATCGCCTGTT<br>AGAGTGACATTCCTCTGGATCTG
	SE	CCTCTTTGTCTTTACGGTTTCC<br>GTCCCAGTGCCTTTGATGTT
	DHCR-7	GCTGCAAAATCGCAACCCAA<br>GCTCGCCAGTGAAAACCAGT
	MSMO-1	TGCTTTGGTTGTGCAGTCATT<br>GGATGTGCATATTCAGCTTCCA
	ApoE	GTTGCTGGTCACATTCCTGG<br>GCAGGTAATCCCAAAAGCGAC
	ACLY	ATCGGTTCAAGTATGCTCGGG<br>GACCAAGTTTTCCACGACGTT
	SREBP-1c	ACAGTGACTTCCCTGGCCTAT<br>GCATGGACGGGTACATCTTCAA
	ACC-1	ATGTCTGGCTTGCACCTAGTA<br>CCCCAAAGCGAGTAACAAATTCT
	SCD	TCTAGCTCCTATACCACCACCA<br>TCGTCTCCAACTTATCTCCTCC
	FASN	CCGAGACACTCGTGGGCTA<br>CTTCAGCAGGACATTGATGCC
	FASD-2	GACCACGGCAAGAACTCAAAG<br>GAGGGTAGGAATCCAGCCATT
	SCD-5	TGGCTGTTTGTTCGCAAGC<br>GGACCACAGGATCAGCAAGC
	ATF6	GCTTTACATTCCTCCACCTCCTTG<br>ATTTGAGCCCTGTTCCAGAGCAC
	PERK	TGCATATAGTGGAAAGGTGAGGT<br>CGAGGTCCGACAGCTCTAAC
	IRE1α	GTACGACACCAAAACCCGAG<br>CGTCCCCAGATTCACTGTCC
	XBP-1	GCAAGCGACAGCGCCT<br>TTTTCAGTTTCCTCCTCAGCG
	GRP78	ACTCCTGAAGGGGAACGTCT<br>ACCACCTTGAACGGCAAGAA
	DR5	AAGACCCTTGTGCTCGTTGT<br>CCAGGTGGACACAATCCCTC

	GRP94	TTCCGCCTTCCTTGTAGCAG<br>AGCTAGGACTCCTCTGGCAA
	CPT1A	ATCAATCGGACTCTGGAAACGG<br>TCAGGGAGTAGCGCATGGT
	HSP90AB1	TTGCCCAACTCATGTCCCTC<br>GTACGTTCCTGAGGGTTGGG
	HSP90AA1	TGGACAGCAAACATGGAGAG<br>CCAGGTGTTTCTTTGCTGCC
